# Ethanolic Extract of Glycine Semen Preparata Prevents Oxidative Stress-Induced Muscle Damage in C2C12 Cells and Alleviates Dexamethasone-Induced Muscle Atrophy and Weakness in Experimental Mice

**DOI:** 10.3390/antiox14070882

**Published:** 2025-07-18

**Authors:** Aeyung Kim, Jinhee Kim, Chang-Seob Seo, Yu Ri Kim, Kwang Hoon Song, No Soo Kim

**Affiliations:** 1 KM Application Center, Korea Institute of Oriental Medicine, Daegu 41062, Republic of Korea; aykim71@kiom.re.kr; 2 KM Convergence Research Division, Korea Institute of Oriental Medicine, Daejeon 34054, Republic of Korea; jinheekim@kiom.re.kr (J.K.); kyuri35@kiom.re.kr (Y.R.K.); 3 KM Science Research Division, Korea Institute of Oriental Medicine, Daejeon 34054, Republic of Korea; csseo0914@kiom.re.kr

**Keywords:** muscle atrophy, Glycine Semen Preparata, fermented black soybean, oxidative stress, glucocorticoid

## Abstract

Skeletal muscle atrophy is a debilitating condition characterized by the loss of muscle mass and function. It is commonly associated with aging, chronic diseases, disuse, and prolonged glucocorticoid therapy. Oxidative stress and catabolic signaling pathways play significant roles in the progression of muscle degradation. Despite its clinical relevance, few effective therapeutic options are currently available. In this study, we investigated the protective effects of an ethanolic extract of Glycine Semen Preparata (GSP), i.e., fermented black soybeans, using in vitro and in vivo models of dexamethasone (Dexa)-induced muscle atrophy. In C2C12 myoblasts and myotubes, GSP significantly attenuated both oxidative stress-induced and Dexa-induced damages by reducing reactive oxygen species levels and by suppressing the expression of the muscle-specific E3 ubiquitin ligases MuRF1 and Atrogin-1. Moreover, GSP upregulated key genes involved in muscle regeneration (*Myod1* and *Myog*) and mitochondrial biogenesis (*PGC1α*), indicating its dual role in muscle protection and regeneration. Oral administration of GSP to mice with Dexa-induced muscle atrophy resulted in improved muscle fiber integrity, increased proportion of large cross-sectional area fibers, and partial recovery of motor function. Isoflavone aglycones, such as daidzein and genistein, were identified as active compounds that contribute to the beneficial effects of GSP through antioxidant activity and gene promoter enhancement. Thus, GSP is a promising nutraceutical that prevents or mitigates muscle atrophy by targeting oxidative stress and promoting myogenesis and mitochondrial function. Further studies are warranted to standardize the bioactive components and explore their clinical applications.

## 1. Introduction

Skeletal muscle atrophy is defined as the reduction in muscle mass and cross-sectional area as a result of decreased muscle protein content, fiber shrinkage, and imbalances in the processes of protein synthesis and degradation [1]. This condition diminishes muscle strength and functional capacity. Skeletal muscle atrophy can be classified according to its primary cause. Disuse atrophy arises from prolonged immobilization, which may occur due to accidents or extended bed rest, as well as exposure to microgravity conditions such as those experienced during spaceflight [2]. In contrast, denervation atrophy is initiated by the loss of neural stimulation, which can result from spinal cord injury, amyotrophic lateral sclerosis, and other conditions [1]. Various illnesses, including cancer, chronic obstructive pulmonary disease, and heart failure, can lead to a specific form of skeletal muscle wasting known as cachexia. Sarcopenia, which is characterized by age-related muscle loss, is primarily attributed to sedentary lifestyles and hormonal changes associated with aging [3]. Furthermore, the chronic use of certain medications, particularly corticosteroids, can induce glucocorticoid-induced atrophy [4].

The World Health Organization assigned sarcopenia an independent disease code (ICD-10 M62.84) in 2016, and the revised Korean Standard Classification of Diseases-8 (KCD-8) also included sarcopenia as a clinical condition in 2021, with diagnostic code M62.5 [5,6]. According to a 2024 study based on the Korean National Health and Nutrition Examination Survey, the prevalence of sarcopenia among Koreans aged 60 years and older is reported to be 6.8%, with rates of 5.5% in men and 7.9% in women. Among those aged 80 years and above, the prevalence increases sharply to 21.5% in men and 25.9% in women [7]. Despite increasing recognition of sarcopenia as a significant health issue, there are currently no approved pharmacological therapies for its treatment either in Korea or globally. Although several investigational agents, including myostatin inhibitors and anabolic compounds, are undergoing clinical trials, none have yet been approved for clinical use [8].

Skeletal muscle atrophy has profound physiological, metabolic, and functional implications that significantly impact overall health and quality of life. The reduction in muscle strength and endurance leads to a decreased ability to produce force, thereby complicating physical activities, such as walking, standing, and lifting. Given that the skeletal muscle serves as the primary site for glucose uptake, skeletal muscle atrophy can lead to decreased insulin sensitivity, thereby increasing the risk of developing type 2 diabetes, hyperglycemia, and metabolic syndrome due to compromised glucose metabolism [9,10]. Furthermore, the skeletal muscle functions as a source of myokines (e.g., interleukin-6 and irisin), which play critical roles in regulating inflammation and immune responses [11]. Consequently, muscle loss diminishes anti-inflammatory signaling and thus exacerbates chronic inflammation and immune suppression, particularly in the context of aging or disease. Importantly, skeletal muscle atrophy is not merely localized; it exerts systemic effects on metabolism, cardiovascular health, immune function, and psychological well-being [2]. Therefore, preventive strategies such as regular exercise, adequate protein intake, and early rehabilitation are essential to mitigate the effects of muscle atrophy and enhance outcomes in aging populations, individuals with chronic diseases, and those experiencing immobilization.

Glycine Semen Preparata (GSP), commonly referred to as fermented black soybeans or “Douchi” in traditional Chinese medicine, has been utilized for centuries [12,13]. In traditional Chinese medicine, GSP is recognized for its cooling properties, which are believed to be effective in dispelling “heat” and alleviating exterior pathogenic factors. This characteristic aligns with its application in the treatment of various conditions, including fever, irritability, and restlessness [14,15]. Soybeans are widely acknowledged as a significant source of plant-based proteins and are processed to obtain diverse products. The preparation of GSP involves the fermentation of black soybeans, a process that may enhance its medicinal properties [13]. GSP is rich in bioactive compounds, including phenolic acids, flavonoids, and isoflavones, and has demonstrated substantial antioxidant effects [14,16]. These compounds reduce oxidative stress by scavenging free radicals, thereby protecting cells from damage and potentially lowering the risk of chronic diseases, such as cardiovascular disease and cancer [17,18]. Research indicates that GSP mitigates inflammation by modulating inflammatory mediators, including cytokines and enzymes such as cyclooxygenase-2, rendering it beneficial in the treatment of arthritis and other inflammatory disorders [19]. Furthermore, studies have suggested that GSP possesses anticancer properties. Fermentation enhances the production of bioactive peptides and isoflavones, such as genistein, which inhibit cancer cell growth, induce apoptosis (programmed cell death), and prevent metastasis [20,21,22]. GSP also exhibits antimicrobial properties by inhibiting the growth of various pathogenic bacteria and fungi. This antimicrobial effect may be attributed to the production of organic acids such as lactic acid during fermentation, which lowers the pH and creates an unfavorable environment for microbial proliferation [23,24]. Some studies have suggested that GSP reduces blood cholesterol levels by decreasing cholesterol absorption in the intestines and increasing bile acid excretion [25]. Additionally, GSP exerts immunomodulatory effects by enhancing macrophagic activity and increasing the production of immune-related cytokines [13,14,19]. Emerging evidence indicates that GSP may have neuroprotective effects, potentially aiding the prevention of neurodegenerative diseases such as Alzheimer’s disease [13,26]. The antioxidants and isoflavones present in GSP may help mitigate oxidative damage to the brain, thereby contributing to improved cognitive function. In summary, GSP exhibits a broad range of biological activities, including antioxidant, anti-inflammatory, anticancer, antimicrobial, and neuroprotective activities. These properties are attributed to its rich biochemical composition, which is enriched during fermentation.

Soy-based products have been extensively researched to determine their potential to mitigate muscle atrophy. Both in vitro and in vivo studies have indicated that soy protein and isoflavones may play significant roles in preserving muscle mass and function [27,28]. Research into various fermented soybean products has provided valuable insights into their effectiveness in promoting muscle health. For instance, “Miso”, a traditional Japanese fermented soybean paste, suppresses muscle atrophy induced by high-fat/high-sucrose diets in murine models by enhancing the gut microbiota and increasing the production of short-chain fatty acids [29]. However, studies on the effects of GSP on muscle health are scarce. This study aimed to determine whether the ethanolic extract of GSP can alleviate skeletal muscle atrophy under oxidative stress and elevated corticosteroid levels. Furthermore, we sought to validate the effectiveness of GSP in an in vivo dexamethasone (Dexa)-induced muscle atrophy model and identify the specific compounds responsible for some of its observed activities.

## 2. Materials and Methods

### 2.1. Preparation of GSP

Freeze-dried GSP extract was prepared and supplied by KOC Biotech (Daejeon, Republic of Korea) following a standardized protocol. Raw GSP plant materials, processed in accordance with the Korean Pharmacopeia, were purchased from Omniherb (Daegu, Republic of Korea). They were mechanically ground and extracted at 85 °C with 70% ethanol at a volume ratio of 10:1 for 3 h using a reflux extraction system (MS-DM609; MTOPS, Seoul, Republic of Korea). The extract was clarified using a cartridge filter with a 5 µm removal rating (SYP05-25, Synopex, Hwaseong, Republic of Korea), concentrated using a rotary evaporator (Ev-1020, SciLab, Seoul, Republic of Korea), and lyophilized in an LP20 freeze dryer (Ilshin-Bio-Base, Dongducheon, Republic of Korea). The yield of the freeze-dried GSP extract was 5.98%. A voucher specimen (KOC-70E-085) was deposited at the KM Convergence Research Division of the Korea Institute of Oriental Medicine (Daejeon, Republic of Korea).

### 2.2. Phytochemical Analysis of GSP via High-Performance Liquid Chromatography (HPLC)

Phytochemical analysis of nine flavonoids (daidzin, glycitin, genistin, acetyldaidzin, acetylglycitin, malonylgenistin, daidzein, glycitein, and genistein) from the GSP extract was performed using a Prominence LC-20A series HPLC system (Shimadzu, Kyoto, Japan) consisting of a solvent delivery unit, online degasser, column oven, autosampler unit, photodiode array detector (PDA), and system controller (LCsolution software, Version 1.24, Shimadzu). These flavonoid compounds were subjected to chromatographic separation using a Capcell Pak UG80 reversed-phase column (250 mm × 4.6 mm, 5 µm, Shiseido, Tokyo, Japan) maintained at 40 °C and gradient elution with a distilled water (solvent A)-acetonitrile (solvent B) mobile phase, with both solvents containing 0.1% (*v*/*v*) formic acid. The gradient elution conditions for the mobile phase were as follows: 10% B (initial time), 25% B (40 min), 90% B (45 min, followed by holding for 10 min), and 10% B (60 min, followed by holding for 10 min). The flow rate and injection volume were 1.0 mL/min and 10 µL, respectively. The target compounds were simultaneously scanned at 190–400 nm using a PDA.

### 2.3. Chemicals

Ascorbic acid (A4544), avertin (T48402), carboxymethylcellulose sodium salt (CMC-Na, C5678), crystal violet solution (V5265), Dexa for in vitro studies (D4902), water-soluble Dexa for animal studies (D2915), dimethyl sulfoxide (DMSO, D8418), hydrogen peroxide (H_2_O_2_) solution (#216763), resveratrol (RsV, R5010), sodium dodecyl sulfate (SDS, L3771), 10% formalin (HT5012), 4′,6-diamidino-2-phenylindole (DAPI, D8417), and tert-amyl alcohol (#152463) were obtained from Sigma-Aldrich (St. Louis, MO, USA). Formic acid (#695076) and acetonitrile (#100030) were obtained from Merck KGaA (Darmstadt, Germany). 1,1-Diphenyl-2-picrylhydrazyl (DPPH, #14805) and 2,2′-azino-bis[3-ethylbenzothiazoline-6-sulfonic acid] (ABTS, A2166) were sourced from Cayman Chemical (Ann Arbor, MI, USA) and Tokyo Chemical Industry (Tokyo, Japan), respectively. Tissue-Tek O.C.T. compound was purchased from Sakura Finetek (Torrance, CA, USA). Ethanol (ER1009-500-00), xylene (XR1014-100-01), and Triton X-100 (TR1020) were purchased from Biosesang (Yongin, Republic of Korea). Harris hematoxylin (#3801562) and alcohol-based eosin (#3801602) solutions were obtained from Leica Biosystems (Wetzlar, Germany). A xylene-based mounting solution (#3801730; Leica Biosystems) was used. Nine flavonoid standard compounds used to assess the quality of GSP extracts using the HPLC system were purchased from high-purity compound manufacturers: daidzin (C_21_H_20_O_9_, CAS No. 552-66-9, purity 99.3%, DR10952) and acetylglycitin (C_24_H_24_O_11_, CAS No. 134859-96-4, purity 99.3%, DR12228) from Shanghai Sunny Biotech (Shanghai, China); glycitin (C_22_H_22_O_10_, CAS No. 40246-10-4, purity 99.6%, CFN99105), malonylgenistin (C_24_H_22_O_13_, CAS No. 51011-05-3, purity 98.1%, CFN90631), and glycitein (C_16_H_12_O_5_, CAS No. 40957-83-3, purity 98.9%, CFN99106) from Wuhan ChemFaces Biochemical (Wuhan, China); genistin (C_21_H_20_O_10_, CAS No. 529-59-9, purity 98.3%, BP0635) from Biopurify Phytochemicals (Chengdu, China); acetyldaidzin (C_23_H_22_O_10_, CAS No. 71385-83-6, purity 95.3%, TBW03633) from ChemNorm Biotech (Wuhan, China); and daidzein (C_15_H_10_O_4_, CAS No. 486-66-8, purity 98.0%, D7802) and genistein from (C_15_H_10_O_5_, CAS No. 446-72-0, purity 98.0%, G6649) from Merck KGaA (Darmstadt, Germany). HPLC-grade solvents (methanol, acetonitrile, and water) were purchased from J. T. Baker (Phillipsburg, NJ, USA).

### 2.4. Cultivation of C2C12 Myoblasts and Their Differentiation into Myotubes

The C2C12 murine myoblast cell line (CRL1772) was obtained from the American Type Culture Collection (Manassas, VA, USA) and cultured in growth medium, specifically Dulbecco’s modified Eagle’s medium (DMEM) containing 4.5 g/L glucose, 10% heat-inactivated fetal bovine serum, and 100 IU penicillin/100 μg/mL streptomycin at 37 °C in a humidified 5% CO_2_ atmosphere. To induce myotube formation, C2C12 myoblasts were placed in growth medium (GM) until confluence and then switched to differentiation medium (DM) consisting of DMEM with 2% heat-inactivated horse serum and 100 IU penicillin/100 μg/mL streptomycin. DM was refreshed every alternate day for 5–7 days. All reagents for cell culture were obtained from Thermo Fisher Scientific (Waltham, MA, USA).

### 2.5. Cytotoxicity Assay for C2C12 Myoblasts

To evaluate the cytotoxic effects of GSP on C2C12 myoblasts, 5000 cells were seeded in each well of a 96-well plate. The cells were treated with GSP or vehicle (0.1% DMSO) after overnight incubation. To investigate the protective effects of GSP under stressful conditions inducing cellular damage, the cells were pretreated with GSP for 12 h and subsequently exposed to 0.25 mM H_2_O_2_ or 0.2 mM Dexa. After 24 h, the supernatants were discarded, and the cells were washed twice with phosphate-buffered saline (PBS; Thermo Fisher Scientific). Adherent cells were analyzed with the EZ-Cytox Enhanced Cell Viability Assay Kit (Daeil Lab Service Co., Ltd., Seoul, Republic of Korea) and a SpectraMax i3 microplate reader (Molecular Devices, LLC, Sunnyvale, CA, USA). The viability of these cells was relatively higher than that of vehicle-treated cells.

### 2.6. Crystal Violet Staining for Assessing Myotube Density

To evaluate the protective effects of GSP and flavonoids against myotube degradation, myotubes differentiated for 5 days were pretreated with GSP, flavonoids, or a vehicle for 12 h, then subsequently treated with 0.25 mM H_2_O_2_ or 0.2 mM Dexa for 24–48 h. After washing with PBS, the cells were stained with a crystal violet solution (0.2% crystal violet containing 20% methanol) for 30 min at 25 °C, washed with tap water, dried, and photographed. To quantify myotube density, the stained cells were dissolved in 1% SDS solution for 30 min at 37 °C, and the absorbance was measured at 590 nm using a SpectraMax i3 microplate reader.

### 2.7. Immunofluorescence Staining

C2C12 myotubes that had been differentiated for 5 days on glass-bottom dishes (SPL Life Sciences, Pocheon, Republic of Korea) were pretreated with GSP for 12 h and then exposed to 0.25 mM H_2_O_2_ for 24 h. After rinsing with PBS, the cells were fixed in 10% neutralized formalin, permeabilized with 0.1% Triton X-100, and blocked with 3% bovine serum albumin (Thermo Fisher Scientific) in PBS for 30 min at 25 °C. After washing, they were incubated overnight at 4 °C with a mouse anti-myosin heavy chain (MyHC) antibody (1:1000 dilution, B4470, R&D Systems, Minneapolis, MN, USA) and then treated with an Alexa Fluor 488-conjugated goat anti-mouse IgG antibody (1:1000 dilution, #A11001, Thermo Fisher Scientific) for 3 h at 25 °C. The cells were counterstained with DAPI, then fluorescent images were captured using a fluorescence microscope (IX71; Olympus Co., Tokyo, Japan). The fusion index and myotube length were assessed in five representative images per group using the ImageJ software, version 1.54f (National Institute of Health, Bethesda, MD, USA). The fusion index was calculated using the following formula: Fusion index (%) = (number of nuclei in multinucleated cells/total number of nuclei) × 100.

### 2.8. Quantitation of Mitochondrial Density

C2C12 myotubes were pretreated with GSP for 12 h, followed by exposure to H_2_O_2_ for 24 h. After the PBS wash, cells were incubated with 50 nM MitoTracker Deep Red FM (M22426; Thermo Fisher Scientific) at 37 °C for 30 min. Mitochondrial red fluorescence was visualized using a fluorescence microscope (Olympus TH4-200, Olympus Optical Co., Tokyo, Japan), and fluorescence intensity was quantified by ImageJ software, version 1.54f.

### 2.9. Promoter Activity Assay

Reporter plasmids for human *Myod1* and *Myog* and murine *PGC1α* promoter were constructed as described in our previous study [26]. GSP- and flavonoid-modulated promoter activities were determined using a luciferase assay system. In brief, C2C12 myoblast cells cultured in 60 mm dishes were transfected with 5.5 µg of each reporter plasmid using Lipofectamine 3000 (Thermo Fisher Scientific). After overnight, cells were trypsinized, resuspended in GM without antibiotics, and replated in a 96-well plate at 25,000 cells/cm^2^. After another 24 h, the cells were exposed to GSP or flavonoids and were additionally incubated for 24 h. After washing the cells with ice-cold PBS, cell lysates were prepared, and intracellular luciferase activity was determined using a commercial luciferase assay system (E1960, Promega, Madison, WI, USA).

### 2.10. Detection of Intracellular Reactive Oxygen Species (ROS) Level

Intracellular ROS in live cells was detected with CellROX™ Green reagent (C10444, Thermo Fisher Scientific). C2C12 myoblasts or differentiation day 5 myotubes were cultured in glass-bottom dishes. The cells were pretreated with GSP or vehicle for 12 h, followed by exposure to 0.25 mM H_2_O_2_ for 6 h. Subsequently, the cells were washed with Hanks’ Balanced Salt Solution (HBSS, #14175, Thermo Fisher Scientific) and incubated with 5 μM CellROX™ Green reagent for 15 min at 37 °C. Fluorescence images of the cells were captured after rewashing the cells with HBSS.

### 2.11. Free Radical-Scavenging Assay

The free radical-scavenging activities of GSP and the flavonoids were determined using ABTS and DPPH assays [30]. For the ABTS assay, the ABTS free radical stock solution was arranged by mixing equal volumes of ABTS (7.4 mM) and potassium persulfate (2.6 mM) solutions for 2 h, protected from light. The ABST working solution was prepared by diluting deep blue ABTS stock solution with water to an optical density of ~0.7 at 734 nm (OD734). Various concentrations of GSP or flavonoids were mixed with equal volumes of the ABTS working solution in a 96-well assay plate (#439454, Thermo Fisher Scientific). The assay mixture was incubated in the dark for 7 min at 25 °C, and the optical density was measured at OD734 using a SpectraMax i3 microplate reader. For the DPPH assay, a working solution was prepared by dissolving 0.2 mM DPPH in absolute methanol in the dark. The assay was initiated by mixing equal volumes of serially diluted GSP or flavonoids with brown DPPH working solutions. The assay mixture was incubated in the dark for 30 min at 25 °C, and then the optical intensity was determined at OD514. Ascorbic acid was used as a positive control in both the ABTS and DPPH assays. The free radical-scavenging activities of GSP, flavonoids, and ascorbic acid were calculated using the formula: inhibition (%) = (1 − OD of GSP, flavonoids, or ascorbic acid/OD of distilled water) × 100.

### 2.12. Induction of Muscle Atrophy and GSP Administration Using C57BL/6N Mice

All animal experiments were performed in accordance with the Guidelines for the Care and Use of Laboratory Animals from the Ministry of Food and Drug Safety, Republic of Korea. The study protocol was approved by the Institutional Animal Care and Use Committee of the Korea Institute of Oriental Medicine (Protocol No. 23-081, approved on 21 August 2023). Nine-week-old male C57BL/6N mice (25–30 g) were purchased from DaeHan BioLink (Samseong-myeon, Republic of Korea) and housed in a specific pathogen-free facility under controlled conditions (22 ± 2 °C, 45 ± 10% humidity, 12/12 h light–dark cycle). Mice had unrestricted access to a standard diet and water. After one week of acclimatization, they were randomly assigned to five groups (*n* = 10/group) and trained for behavioral assessments on day 0 (D0). Water-soluble Dexa was dissolved in PBS and filtered through a 0.22 µm syringe filter (#17761-ACK, Sartorius Korea, Seongnam, Republic of Korea). GSP and RsV were resuspended in sterile 0.5% CMC-Na in PBS. GSP and positive control solutions were stored at −80 °C until use. Starting on experimental day 1 (D1), the mice received oral GSP at 100 mg/kg (group 3) or 300 mg/kg (group 4) daily for 10 days. The positive control group (group 5) received 200 mg/kg RsV, whereas groups 1 and 2 received 0.5% CMC-Na in PBS. Thirty minutes after oral administration, the mice were intraperitoneally injected with sterile PBS (group 1) or 25 mg/kg Dexa (groups 2–5) following established protocols.

### 2.13. Grip Strength Test and Rotarod Test

On experimental day 9 (D9), a grip strength test was conducted with a grip strength meter (DJ-356, Daejong Instrument Industry Co., Seoul, Republic of Korea) to evaluate muscle strength in mice. The mice were placed on a wire grid, and their grip strength was measured by pulling them back by their tails until they released the wire. This was repeated five times for each mouse, and the average was calculated. On experimental day 10 (D10), motor coordination and balance were evaluated using ROTA-ROD (B.S. Technolab Inc., Seoul, Republic of Korea). Initially, the mice were acclimated to the rod for 30 s to allow walking and balancing. The rotational speed of the rod was then increased according to the following program: holding at 4 rpm for 1 min, followed by gradual acceleration from 4 to 40 rpm over 5 min and maintenance at 40 rpm for 5 min. The time until the mouse fell off the rod or exhibited repeated passive rotation was recorded, and the average time of three trials was calculated.

### 2.14. Isolation of Muscle Tissue

On experimental day 11 (D11), the mice were euthanized by intraperitoneal injection of 2% avertin in PBS at 240 mg/kg. Gastrocnemius (GN) and tibialis anterior (TA) muscle tissues were excised from hind limbs and weighed. The tissues were snap-frozen in a dry ice–acetone bath for 10 s, transferred to pre-cooled 1.8 mL cryogenic tubes (#375418PK, Thermo Fisher Scientific) containing cotton pre-wetted with PBS to minimize tissue dryness, and stored at −80 °C for histological analysis.

### 2.15. Cryosectioning and Hematoxylin and Eosin (H&E) Staining of Muscle Tissues

Prior to cryosectioning, the cryostat (CM3050S, Leica Biosystems) and blade were pre-cooled to −22 ± 2 °C. Frozen tissues were equilibrated within the cryostat chamber for 10 min, affixed to metallic holders with O.C.T. compound, and rapidly frozen on dry ice. A 30 µm thick section was obtained on a positively charged slide pre-equilibrated at 22–24 °C and stored at −80 °C. For H&E staining, the slides were equilibrated at 22–24 °C for 30 min and stained with hematoxylin for 10 min. After rinsing with running tap water, they were counterstained with eosin for 1 min and washed thoroughly with distilled water. They were then dehydrated using ethanol with serially increasing concentrations: 70% for 1 min, 80% for 1 min, 90% for 1 min, and 100% for 1 min. Subsequently, the slides were cleared in a 1:1 solution of ethanol and xylene for 3 min and immersed in 100% xylene for 3 min, then mounted with a xylene-based mounting solution and allowed to dry overnight under a fume hood. Images were captured using a BX43 microscope with a DP73 digital camera (Olympus, Tokyo, Japan), and the cross-sectional area (CSA) of more than 100 muscle fibers was quantified using ImageJ software, version 1.54f.

### 2.16. Western Blotting

Total protein was extracted from cells or frozen tissues using M-PER™ Mammalian Protein Extraction Reagent (#78501, Thermo Fisher Scientific) or T-PER™ Tissue Protein Extraction Reagent (#78510, Thermo Fisher Scientific), both of which contain Halt™ Protease and Phosphatase Inhibitor Cocktail (#78444, Thermo Fisher Scientific). Tissues were homogenized using the PreCellys instrument (Bertin Instruments, Montigny-le-Bretonneux, France), then centrifuged at 13,000 rpm for 15 min at 4 °C. Clear lysates were analyzed to determine the protein concentration using bicinchoninic acid assay (#23227, Thermo Fisher Scientific). Proteins of equal amounts were separated by SDS-PAGE and transferred to an Immobilon-P PVDF membrane (IPVH00010, Millipore, Bedford, MA, USA). After blocking the membrane with EzBlock Chemi Solution (AE-1475, ATTO Korea, Daejeon, Republic of Korea), it was incubated overnight at 4 °C with a primary antibody (1:1000 dilution). After rinsing the membrane with 0.1% Tween 20 detergent in Tris-buffered saline, it was treated with horseradish peroxidase (HRP)-conjugated secondary antibody (1:4000 dilution) for 1 h at 25 °C. Target proteins were detected using SuperSignal West Femto Maximum Sensitivity Substrate (#34096; Thermo Fisher Scientific) and ImageQuant LAS4000 Mini (GE Healthcare, Piscataway, NJ, USA). Protein levels were quantified using ImageJ software, version 1.54f after normalization to β-actin or glyceraldehyde 3-phosphate dehydrogenase levels. The antibodies employed in this study included anti-MyHC (MAB4470), anti-muscle atrophy F-box (MAFbx/Atrogin-1, ab168371), anti-phosphorylated FoxO3 (p-FoxO3; s253, ab154786), heme oxygenase 1 (HMOX1, ab13248), glutathione peroxidase 1 (GPX1, ab22604), superoxide dismutase 1 (SOD1, ab51254), peroxisome proliferator-activated receptor gamma coactivator 1-alpha (PGC1α, 66369-lg), anti-muscle ring-finger protein-1 (MuRF1, sc-398608), anti-β-actin (sc-47778), myogenin (MYOG, sc-52903), MYOD1 (sc-32758), and anti-glyceraldehyde 3-phosphate dehydrogenase (GAPDH, sc-32233), sourced from R&D Systems, Abcam (Cambridge, UK), Proteintech (Rosemont, IL, USA), and Santa Cruz Biotechnology (Santa Cruz, CA, USA), respectively. Antibodies against nuclear factor-like 2 (NRF2, #12721), phosphorylated Akt (p-Akt; s473, #9271), Akt (#4685), phosphorylated mTOR (p-mTOR; s2448, #2971), mTOR (#2983), FoxO3 (#9467), HRP-conjugated anti-rabbit IgG (#7074), and anti-mouse IgG (#7076) were purchased from Cell Signaling Technology (Danvers, MA, USA).

### 2.17. Statistical Analysis

Data analysis was performed using GraphPad Prism (version 9.5.1, GraphPad Software, San Diego, CA, USA). The results are presented as the mean ± standard error of the mean (SEM) derived from multiple trials. Group differences were evaluated using one-way analysis of variance followed by Dunnett’s multiple comparison test, with statistical significance set at *p* < 0.05.

## 3. Results

### 3.1. GSP Protects C2C12 Myoblasts from Cytotoxic Conditions and Enhances Promoter Activity

**Figure 1:**
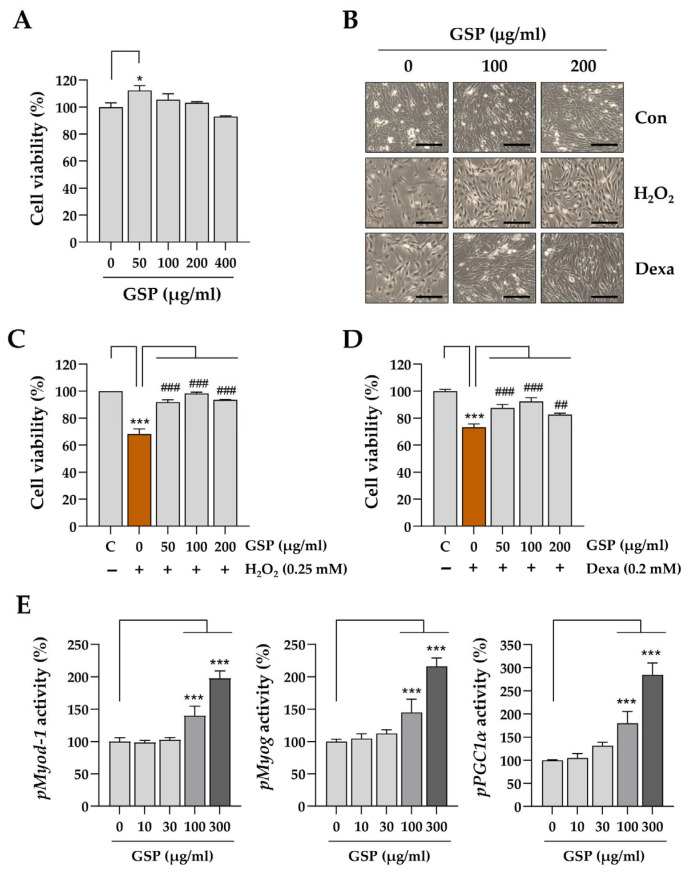
Protective effects of GSP against cytotoxic conditions in C2C12 myoblasts. (**A**) C2C12 myoblasts were treated with varying concentrations of GSP (0–400 μg/mL) for 24 h, after which cell viability was assessed. The cell viability, relative to vehicle-treated controls, is presented as the mean ± SEM (*n* = 3). (**B**) Myoblasts were pretreated with 100 or 200 μg/mL of GSP or vehicle for 12 h and then exposed to 0.25 mM H_2_O_2_ or 0.2 mM Dexa for 24 h. Morphological changes were observed using an inverted microscope. (**C**,**D**) Myoblasts pretreated with GSP or vehicle were subsequently treated with 0.25 mM H_2_O_2_ (**C**) or 0.2 mM Dexa (**D**) for an additional 24 h. Cell viability was measured, and the relative values are expressed as means ± SEM (*n* = 3). (**E**) Reporter plasmids for *Myod1*, *Myog*, and *PGC1α* promoters were transfected into C2C12 myoblast cells, which were subsequently treated with GSP at concentrations ranging from 0 to 300 μg/mL for 24 h. The promoter activity was assessed using a luciferin substrate reaction, as detailed in the Materials and Methods, and the relative values are expressed as means ± SEM (*n* = 3). * *p* < 0.05, *** *p* < 0.001 vs. vehicle-treated controls; ## *p* < 0.01, ### *p* < 0.001 vs. cytotoxic condition + vehicle-treated controls. Scale bar = 100 μm. Dexa, dexamethasone; GSP, Glycine Semen Preparata.

To evaluate the effects of GSP on muscle cells, we assessed the viability of C2C12 myoblasts after treating them with increasing GSP concentrations. As shown in Figure 1A, GSP did not induce any cytotoxicity in myoblasts at concentrations of up to 400 μg/mL. Furthermore, treatment with 0.1% DMSO as a vehicle did not result in cytotoxicity, as evidenced by cell viability and morphology (Figure 1B). To induce cytotoxic stress in myoblasts, we employed 0.25 mM H_2_O_2_ as an oxidative stress inducer and 0.2 mM Dexa as a hypercortisolism, in accordance with previous studies [30]. To evaluate the protective effects of GSP under stress conditions, myoblasts were pretreated with either GSP or vehicle for 12 h, followed by exposure to stress conditions for an additional 24 h. As shown in Figure 1B, both H_2_O_2_ and Dexa significantly inhibited myoblast proliferation. However, cells pretreated with GSP exhibited reduced cytotoxic damage. Following treatment with H_2_O_2_ or Dexa, myoblast viability decreased by approximately 32% and 28%, respectively. In contrast, GSP pretreatment maintained cell viability at levels comparable to those of the normal control cells, even in the presence of H_2_O_2_ and Dexa (Figure 1C,D). These findings suggest that GSP protects myoblasts against oxidative stress and glucocorticoid-induced cytotoxicity. Subsequently, we investigated the effects of GSP on the promoter activity of genes associated with muscle cell differentiation and mitochondrial energy metabolism, specifically *Myod1*, *Myog*, and *PGC1α*. C2C12 myoblasts were transfected with each promoter–reporter plasmid and treated with various GSP concentrations for 24 h. The results indicated that GSP increased the promoter activities of *Myod1*, *Myog*, and *PGC1α* in muscle cells in a dose-dependent manner and showed significant enhancement at ≥100 µg/mL (Figure 1E). The effects of GSP on the expression of *Myod1*, *Myog*, and *PGC1α* were further examined by quantitative real-time PCR, and their protein levels were determined using immunoblotting. GSP treatment increased the expression of *Myod1* and *Myog* at both the mRNA and protein levels, whereas *PGC1α* was upregulated only at the protein level (Supplementary Figure S1).

### 3.2. GSP Inhibits Stress-Induced Degradation of C2C12 Myotubes

**Figure 2:**
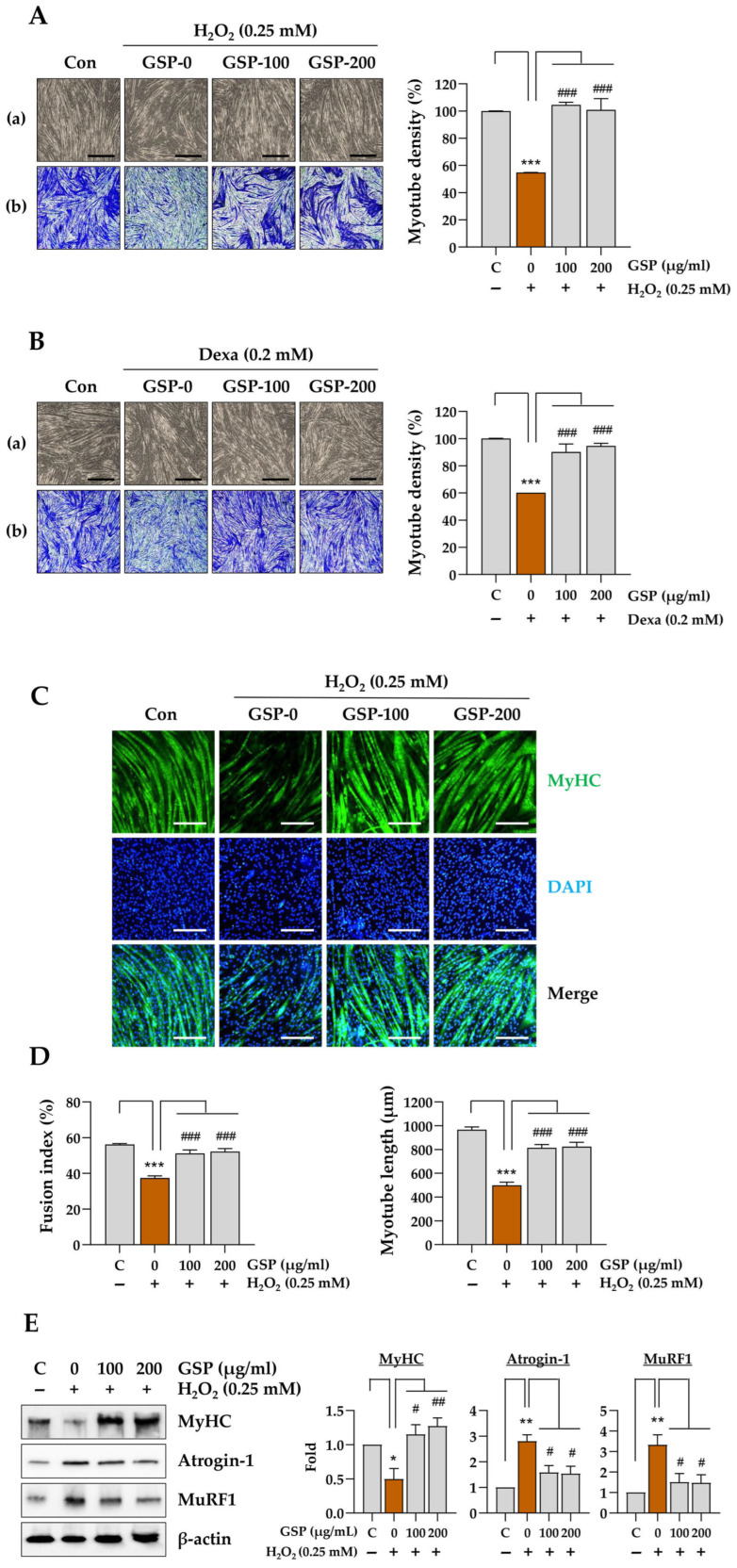

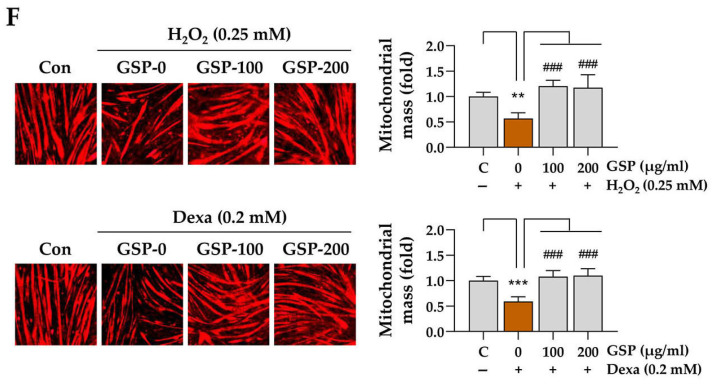
Protective effects of GSP against stressful conditions in C2C12 myotubes. C2C12 myotubes at differentiation day 5 (DD5) were pretreated with 100 and 200 μg/mL of GSP for 24 h and subsequently exposed to 0.25 mM H_2_O_2_ (**A**) or 0.2 mM Dexa (**B**) for an additional 40 h. Morphological changes were observed using an inverted microscope (a) and subsequently assessed through crystal violet staining to quantify myotube density (b). Following solubilization with 1% SDS, myotube density was quantified as described previously. Relative values, compared to those of vehicle-treated control cells, are presented as means ± SEM (*n* = 3). (**C**) Myotubes were pretreated with GSP for 12 h and then treated with 0.25 mM H_2_O_2_. After 24 h, cells were subjected to immunofluorescent staining for MyHC (green) and DAPI counterstaining (blue). (**D**) Fusion index and myotube length were quantitated and presented as means ± SEM (*n* = 6–11). (**E**) Protein levels of myosin heavy chain (MyHC), Atrogin-1, and MuRF1 in C2C12 myotubes were quantified using immunoblotting. β-actin served as a loading control. (**F**) Myotubes were pretreated with GSP for 12 h and further incubated with H_2_O_2_. After 24 h, mitochondrial mass was detected using MitoTracker Deep Red dye. The data are expressed as means ± SEM (*n* = 3). * *p* < 0.05, ** *p* < 0.01, *** *p* < 0.001 vs. vehicle-treated controls; # *p* < 0.05, ## *p* < 0.01, ### *p* < 0.001 vs. stress condition + vehicle-treated controls. Scale bar = 100 μm. Dexa, dexamethasone; GSP, Glycine Semen Preparata.

To evaluate the protective effects of GSP against stress-induced degradation of C2C12 myotubes, the cells were pretreated with GSP at concentrations of 100 and 200 µg/mL for 24 h. Following pretreatment, the cells were exposed to either 0.25 mM H_2_O_2_ or 0.2 mM Dexa for an additional 40 h. As shown in Figure 2A,B, H_2_O_2_ and Dexa induced myotube degradation, characterized by the collapse and shortening of their morphology (a), which was more distinctly observed by crystal violet staining (b). Quantitative analysis indicated that the myotube density decreased by approximately 45.2% following H_2_O_2_ treatment and 39.8% following Dexa treatment compared to vehicle treatment. In contrast, GSP pretreatment significantly alleviated myotube degradation and restored myotube density under these stress conditions. Immunofluorescence staining revealed elevated levels of MyHC (green fluorescence), a recognized marker of muscle differentiation characterized by the presence of more than ten nuclei. H_2_O_2_ treatment resulted in significantly reduced MyHC expression; however, pretreatment with GSP effectively countered this decline, maintaining MyHC levels comparable to those observed in control myotubes (Figure 2C). Control myotubes typically exhibited a fusion index of approximately 56.3%. In contrast, H_2_O_2_ treatment led to a 33.4% reduction in fusion index and approximately 4a 8.4% decrease in myotube length. Notably, pretreatment with GSP preserved the fusion index at a level similar to that of control myotubes and maintained the myotube length at 85% of that in control myotubes, even in the presence of H_2_O_2_ (Figure 2D). Furthermore, immunoblotting analysis demonstrated that H_2_O_2_ treatment significantly decreased MyHC protein expression in myotubes while concurrently increasing the levels of the muscle-degrading proteins Atrogin-1 and MuRF1. Importantly, GSP pretreatment effectively mitigated H_2_O_2_-induced downregulation of MyHC and upregulation of Atrogin-1 and MuRF1 (Figure 2E). To determine whether these muscle-protective effects were associated with enhanced mitochondrial function, we measured mitochondrial content using MitoTracker Deep Red staining. As shown in Figure 2F, GSP pretreatment led to a significant increase in mitochondrial mass compared to the H_2_O_2_ group, supporting the notion that GSP-mediated activation of *PGC-1α* contributes to mitochondrial preservation and biogenesis under oxidative stress conditions. Collectively, these findings suggested that GSP exhibits protective potential against H_2_O_2_-induced muscle atrophy through mitochondrial preservation.

### 3.3. GSP Reduces Oxidative Stress in H_2_O_2_-Treated C2C12 Cells and Exhibits ROS-Scavenging Activity

**Figure 3:**
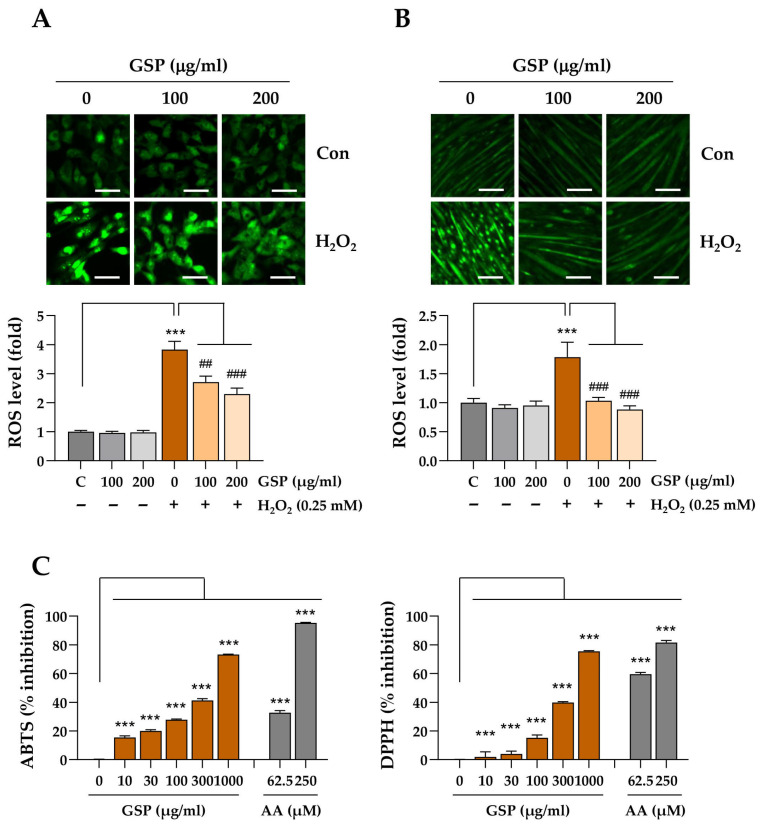
Antioxidant activity of GSP. (**A**,**B**) C2C12 myoblasts (**A**) and myotubes (**B**) were pretreated with GSP (0, 100, and 200 μg/mL) for 12 h and then exposed to 0.25 mM H_2_O_2_ for 6 h. Intracellular oxidative stress was evaluated using ROS-sensitive CellROX™ Green dye and visualized under a fluorescent microscope. The fold increase in ROS for each treatment group was compared to vehicle-treated controls. Data are presented as means ± SEM (*n* = 3). (**C**) Free radical-scavenging activities of GSP were determined using ABTS and DPPH assays. Ascorbic acid was included in parallel as a positive control in both assays. Data are presented as means ± SEM (*n* = 2). *** *p* < 0.001 vs. vehicle-treated controls; ## *p* < 0.01, ### *p* < 0.001 vs. H_2_O_2_ + vehicle-treated controls. Scale bar = 100 μm. AA, ascorbic acid; Dexa, dexamethasone; GSP, Glycine Semen Preparata.

To examine the effects of GSP on intracellular ROS production in C2C12 myoblasts (Figure 3A) and myotubes (Figure 3B), C2C12 myoblasts were pretreated with 100 and 200 µg/mL GSP prior to H_2_O_2_ treatment. Subsequently, the cells were labeled with ROS-sensitive CellROX^TM^ green dye. No significant changes in intracellular ROS levels were detected in the myoblasts and myotubes treated with GSP alone. However, following H_2_O_2_ treatment, both myoblasts and myotubes displayed intense green fluorescence, indicating elevated ROS production inside the cells. In contrast, GSP pretreatment resulted in significantly reduced green fluorescence intensity in a dose-dependent manner. To further investigate the antioxidant potential of GSP using a cell-free biochemical assay, the free radical-scavenging activities of GSP were evaluated through the established biochemical methods, ABTS and DPPH assays. As shown in Figure 3C, GSP eliminated ABTS and DPPH free radicals in a dose-dependent manner. The inhibitory concentrations 50% (IC_50_) of GSP calculated from the ABTS and DPPH assays were 362 µg/mL and 429 µg/mL, respectively. Ascorbic acid, which served as a positive control in both assays, exhibited dose-dependent free radical-scavenging activities, with IC_50_ values of 89 µM and 48 µM in the ABTS and DPPH assays, respectively. Collectively, these findings suggested that GSP effectively mitigates oxidative stress, thereby protecting cells against oxidative stress-induced cytotoxicity. The effect of GSP on the expression of genes associated with antioxidant activity was evaluated in C2C12 myoblasts. Treatment with GSP alone led to an increase in the protein levels of NRF2 and HMOX1. In contrast, GPX1 expression was downregulated by GSP, while SOD1 levels remained unaffected (Supplementary Figure S2). Under oxidative stress induced by H_2_O_2_, genes involved in the Nrf2 signaling pathway, including *Nfe2l2*, *Nqo1*, *Hmox1*, and *Cat*, were significantly upregulated in both C2C12 myoblasts and myotubes. *Sod1* expression also showed a statistically significant increase, albeit to a lesser extent (Supplementary Figure S3). Under H_2_O_2_-induced oxidative stress conditions, C2C12 cells exhibited differential responses to GSP pretreatment. In myoblasts, GSP pretreatment resulted in a modest further increase in *Nqo1* and *Sod1* expression, whereas *Hmox1* was significantly downregulated (Supplementary Figure S3A). In differentiated C2C12 myotubes, *Nqo1* and *Sod1* were further increased, while *Hmox1*, *Cat*, and *Gpx1* were reduced by GSP pretreatment (Supplementary Figure S3B).

### 3.4. Oral Administration of GSP Improves Muscle Function and Alleviates Muscle Loss in Experimental Mice with Dexa-Induced Muscle Atrophy

**Figure 4:**
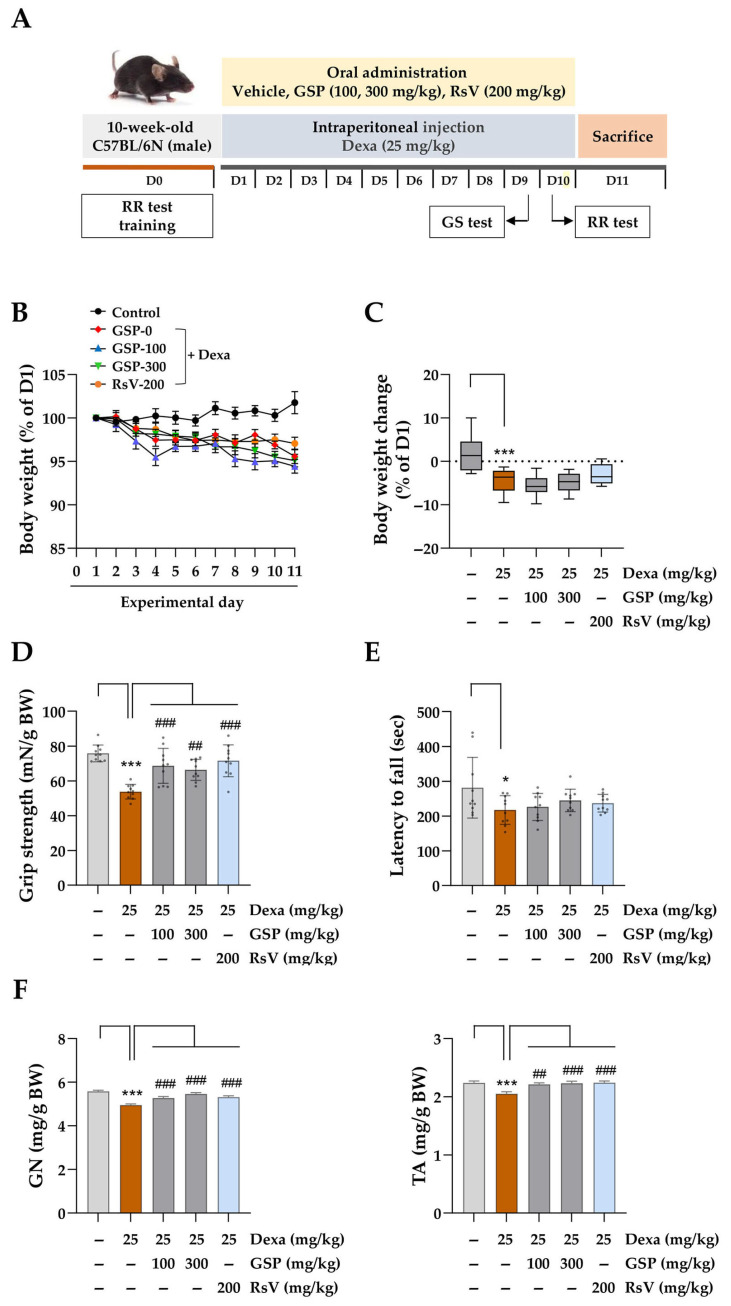
Effects of oral GSP administration in mice with Dexa-induced muscle atrophy. (**A**) The experimental schedule, including the induction of muscle atrophy, GSP and positive control administration, and behavioral evaluation, is outlined. (**B**) Body weight was recorded daily throughout the experimental period, and relative body weight was determined by comparison with the body weight on day 1 (D1). The data are presented as means ± SEM (*n* = 10). (**C**) The change in body weight on day 11 (D11) was determined by comparing it to the weight recorded on D1. The dashed line indicates no change in body weight compared to day 1 (baseline, 0%). Data are presented as means ± SEM (*n* = 10). (**D**) Grip strength tests were conducted on D9 and normalized by body weight. Data are expressed as means ± SEM (*n* = 10). Dots indicate individual values. (**E**) On day 10 (D10), the rotarod test was conducted to evaluate motor coordination capabilities by measuring the time until the mice fell off the rotating rod. Data are expressed as means ± SEM (*n* = 10). Dots indicate individual values. (**F**) On day 11 (D11), the mice were euthanized, and the gastrocnemius (GN) and tibialis anterior (TA) muscles were excised and weighed. Muscle weight was normalized to body weight and reported as means ± SEM (*n* = 10). * *p* < 0.05, *** *p* < 0.001 vs. vehicle-treated group, ## *p* < 0.01, ### *p* < 0.001 vs. Dexa + vehicle-treated group. Dexa, dexamethasone; GSP, Glycine Semen Preparata.

Up to this point, we have demonstrated the protective effects of GSP against stressful conditions induced by oxidative stress and high concentrations of Dexa using in vitro models of muscle atrophy. Previous studies, including our own, have shown that chronic administration of Dexa induces muscle atrophy in experimental animals, accompanied by oxidative stress [31,32]. To investigate the impact of GSP on Dexa-induced muscle atrophy in murine models, GSP was administered daily before Dexa injection, and body weights were monitored throughout the study. RsV was used as a positive control because of its established efficacy in mitigating Dexa-induced muscle loss, as demonstrated by both in vitro and in vivo studies (Figure 4A) [30,32,33]. Vehicle-treated control mice exhibited progressive increases in body weight. In contrast, mice receiving daily Dexa injections exhibited a sustained decrease in body weight throughout the experimental period, corroborating the findings of a previous study [30,32,33]. Importantly, oral GSP administration did not mitigate Dexa-induced weight loss during the experimental period (Figure 4B). The change in body weight relative to the initial measurement (D1) was calculated on the final day of this study (Figure 4C). The body weight of normal control mice increased slightly, reaching 101.77% of the body weight recorded on D1. In contrast, mice-administered GSP at 100 mg/kg (95.45%) and 300 mg/kg (95.01%) demonstrated no significant changes in body weight compared with those receiving Dexa alone (95.58%). Mice treated with RsV exhibited a slight increase in body weight (97.06%); however, this change was not statistically significant. To examine the effects of GSP on muscle strength and physical performance in mice with Dexa-induced muscle atrophy, forelimb grip strength and rotarod performance were evaluated on D9 and D10, respectively. As shown in Figure 4D, mice treated with Dexa exhibited significantly reduced grip strength, which was approximately 29.1% lower than that in the control group. In contrast, co-administration of GSP at 100 mg/kg and 300 mg/kg significantly restored grip strength to 90.5% and 87.5% of that of control mice, respectively. Furthermore, RsV treatment significantly improved grip strength, which was consistent with that of the control mice. In the rotarod test, which assesses motor coordination, mice receiving only Dexa fell off the rod earlier than control mice. Mice treated with 300 mg/kg GSP or RsV exhibited longer latency before falling, although the difference was not statistically significant (Figure 4E). These behavioral assessments provide strong evidence that GSP potentially mitigates muscle dysfunction induced by chronic exposure to Dexa in experimental mice. Given that Dexa-induced muscle dysfunction was mitigated by GSP, we further examined whether this protective effect was associated with the inhibition of muscle loss and muscle atrophy. As shown in Figure 4F and Supplementary Figure S4, Dexa administration reduced the weights of the GN and TA muscles. Conversely, the co-administration of GSP and RsV promoted the recovery of the weight of both muscles.

### 3.5. Oral Administration of GSP Mitigates Dexa-Induced Muscle Atrophy and Degradation

**Figure 5:**
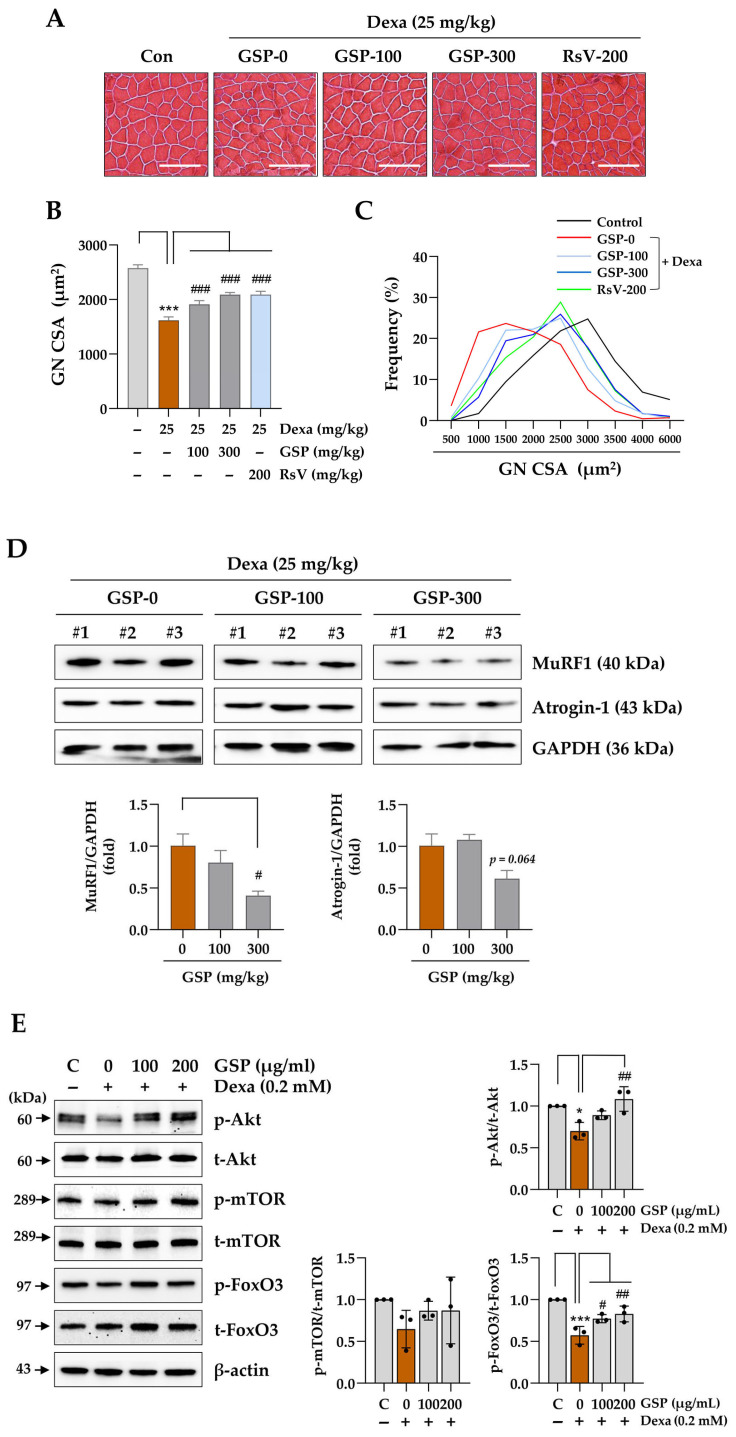
Effects of oral GSP administration on the myofiber integrity and expression of muscle degradation-related proteins in muscle tissues of mice with Dexa-induced muscle atrophy. (**A**) To assess muscle atrophy, gastrocnemius (GN) muscle tissue sections were stained with H&E and examined under a microscope. Images depicting cross-sections of myofibers are presented. Scale bar = 100 μm. (**B**) The cross-sectional area (CSA) of the GN muscle fibers was quantified using ImageJ software, version 1.54f and expressed as means ± SEM (*n* = 5). A minimum of 500 myofibers were measured in each sample. (**C**) The frequency distribution of the CSA of myofibers was analyzed. (**D**) The MuRF1 and Atrogin-1 protein levels in GN muscles were determined by immunoblotting (*n* = 3). GAPDH served as a loading control. (**E**) C2C12 myotubes at differentiation day 5 (DD5) were pretreated with 100 and 200 μg/mL of GSP for 15 h and subsequently exposed to 0.2 mM Dexa for 24 h. Protein levels in C2C12 myotubes were quantified using immunoblotting. β-actin served as a loading control. The data are expressed as means ± SEM (*n* = 3). Dots indicate individual values. * *p* < 0.05, *** *p* < 0.001 vs. vehicle-treated group, # *p* < 0.05, ## *p* < 0.01, ### *p* < 0.001 vs. Dexa + vehicle-treated group. CSA, cross-sectional area; Dexa, dexamethasone; GN, gastrocnemius; GSP, Glycine Semen Preparata.

To evaluate the effects of GSP on myofiber size, muscle sections were stained with H&E, and the CSA was measured by following an established protocol [30]. Representative images of the cross-section of myofibers in the GN and TA are presented in Figure 5A and Supplementary Figure S5A, respectively. Dexa injection reduced the mean CSA of the GN and TA muscles by approximately 37.1% and 23.3%, respectively, compared to those in control mice. Co-administration of 300 mg/kg GSP restored the CSA of the GN and TA to 81.1% and 112.8% of the control levels, respectively. Furthermore, RsV administration restored the CSA of the GN and TA to 81.2% and 104.2% of the control levels, respectively (Figure 5B, Supplementary Figure S5B). In the control group, the proportion of CSA distribution in GN was approximately 27.0% and 73.0% for the ranges of 500–2000 μm^2^ and 2000–6000 μm^2^, respectively. Following Dexa injection, these proportions shifted to approximately 70.5% and 29.5%, respectively. In the groups receiving 100 and 300 mg/kg GSP, the proportion of CSA distribution in the 500–2000 μm^2^ range was approximately 55.3% and 46.07%, respectively, while for the 2000–6000 μm^2^ range, it was about 44.7% and 53.9%, respectively. The group-administered RsV demonstrated a CSA distribution pattern similar to that of the control group, with 43.8% in the 500–2000 μm^2^ range and 56.2% in the 2000–6000 μm^2^ range (Figure 5C). The control group exhibited a CSA distribution proportion of 57.4% within the 200–1200 μm^2^ range and 42.8% within the 1200–3000 μm^2^ range in the TA. After Dexa administration, these proportions increased to 75.6% and 24.4%, respectively. In contrast, the groups co-treated with GSP and RsV demonstrated a distribution pattern similar to that of the control group, with values ranging from 52.2% to 56.9% in the 200–1200 μm^2^ range and from 43.1% to 47.8% in the 1200–3000 μm^2^ range (Supplementary Figure S5C). We also investigated the protective effects of GSP against Dexa-induced muscle loss by examining its effects on muscle degradation-related proteins. Dexa treatment led to increased levels of Atrogin-1 and MuRF1 in mice, whereas co-administration of GSP led to moderately decreased levels of both proteins (Figure 5D and Supplementary Figure S5D). These findings indicate that GSP may confer protective benefits against Dexa-induced muscle atrophy, which is related to enhanced muscle function, including grip strength. To elucidate the molecular mechanisms underlying the protective effects of GSP against muscle atrophy, we investigated the Akt/mTOR/FoxO3 signaling pathway, which is a well-characterized regulator of Dexa-induced muscle atrophy, in C2C12 myotubes. Western blot analysis revealed that Dexa treatment significantly reduced the phosphorylation levels of Akt, mTOR, and FoxO3, indicating the activation of catabolic signaling. Notably, pretreatment with GSP effectively restored the phosphorylation of these key molecules, thereby counteracting the Dexa-induced suppression of the Akt/mTOR/FoxO3 axis (Figure 5E). These findings suggest that the anti-atrophic effects of GSP are at least partially mediated through the reactivation of the Akt/mTOR/FoxO3 signaling pathway.

### 3.6. HPLC–PDA Profiling Analysis of GSP and Identification of Flavonoids That Contribute to Protection Against Muscle Damage in C2C12 Cells

**Figure 6:**
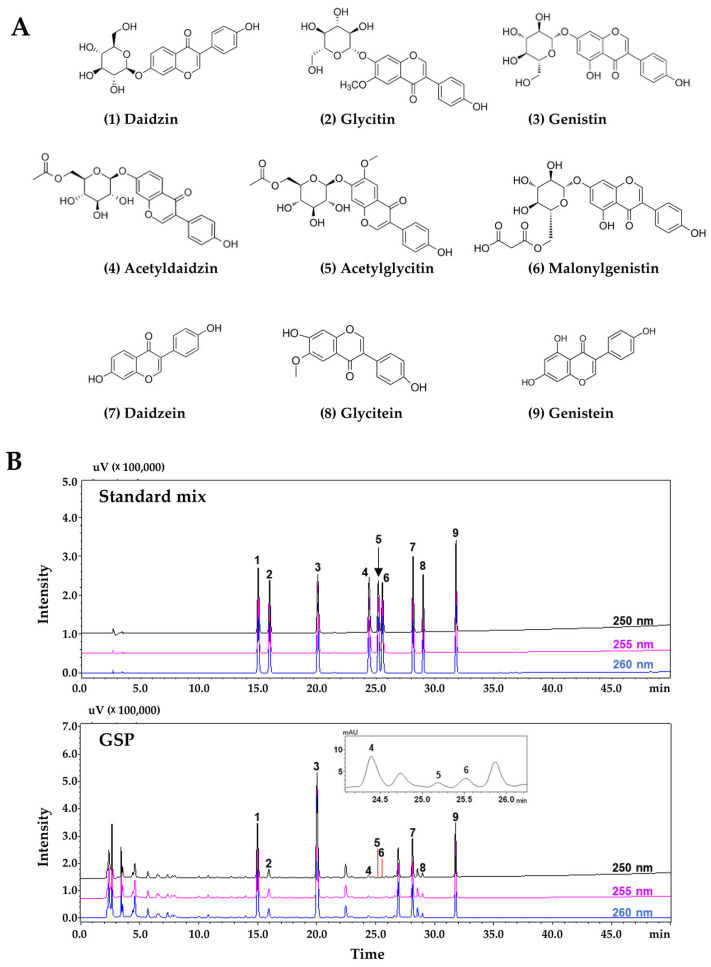

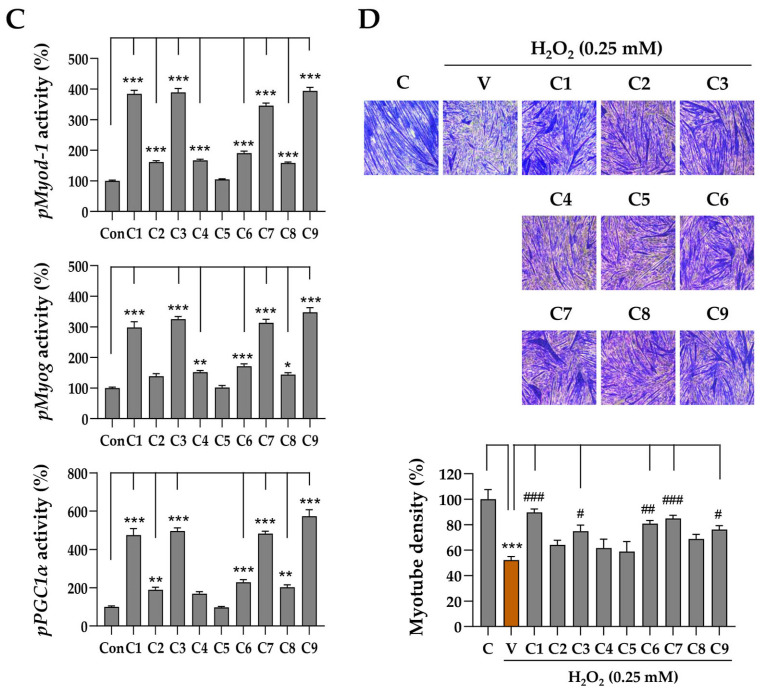
HPLC analysis of GSP and identification of flavonoids exhibiting muscle-protective effects. (**A**) Chemical structure of nine flavonoids. (**B**) HPLC chromatograms of standard solutions and GSP samples. Daidzin (1), glycitin (2), genistin (3), acetyldaidzin (4), acetylglycitin (5), malonylgenistin (6), daidzein (7), glycitein (8), and genistein (9). (**C**) Reporter plasmids for *Myod1*, *Myog*, and *PGC1α* were transfected into C2C12 myoblasts and treated with 20 µM of each flavonoid for 24 h. Promoter activity was measured using a luciferin substrate reaction. (**D**) C2C12 myotubes on differentiation day 5 were pretreated with 20 µM of each flavonoid for 12 h and then exposed to 0.25 mM H_2_O_2_ for 24 h. Myotube density was quantified using crystal violet staining and 1% SDS solubilization; the results are presented as means ± SEM (*n* = 3) compared to vehicle-treated controls. All microscopic images were captured at ×100 magnification. * *p* < 0.05, ** *p* < 0.01, *** *p* < 0.001 vs. vehicle-treated controls; # *p* < 0.05, ## *p* < 0.01, ### *p* < 0.001 vs. H_2_O_2_ + vehicle-treated controls. GSP, Glycine Semen Preparata.

The optimized HPLC–PDA analysis conditions were successfully applied for the simultaneous determination of nine flavonoids (daidzin, glycitin, genistin, acetyldaidzin, acetylglycitin, malonylgenistin, daidzein, glycitein, and genistein; Figure 6A) selected for quality control of GSP. They were completely eluted within 35 min without interference from components representing the neighboring peaks. The detection wavelength for the quantitative analysis of each compound was as follows: 250 nm for daidzin, acetyldaidzin, and daidzein; 255 nm for glycitin and glycitein; and 260 nm for genistin, acetylglycitin, malonylgenistin, and genistein (Figure 6B). Using the established analysis method, nine flavonoids were detected in the GSP sample at 0.10–8.07 mg/g of freeze-dried GSP (Table 1). Among these compounds, genistin was the most abundant at 8.07 mg/g GSP. To elucidate the components that contribute to the muscle-protective effects of GSP, we investigated the impact of flavonoids (C1–C9) on the promoter activity of *Myod1*, *Myog*, and *PGC1α* in C2C12 myoblasts at 20 μM. As shown in Figure 6C, the flavonoids daidzin (C1), genistin (C3), daidzein (C7), and genistein (C9) significantly enhanced the promoter activity of *Myod1*, *Myog*, and *PGC1α*, resulting in more than a threefold increase. Furthermore, in C2C12 myotubes, pretreatment with daidzin (C1), genistin (C3), malonylgenistin (C6), daidzein (C7), and genistein (C9) effectively mitigated H_2_O_2_-induced damage (Figure 6D).

## 4. Discussion

Skeletal muscle atrophy results from an imbalance between the rates of muscle protein synthesis and degradation. It is frequently associated with natural aging (sarcopenia), malnutrition, prolonged disuse, and, most importantly, pathological conditions such as diabetes, obesity, cachexia, and Alzheimer’s disease [2,3,34,35,36]. Muscle atrophy can lead to poor quality of life, increased risk of fractures, and reduced basal metabolic rate [37]. To date, Food and Drug Administration-approved treatments for muscle atrophy, such as nusinersen and risdipam, are primarily used for specific muscle-related conditions, such as spinal muscular atrophy [38]; however, limited medications are available for preventing or treating general muscle loss or muscle atrophy. This study provides compelling and robust evidence that an ethanolic extract of GSP, which is prepared from fermented black soybeans, exerts protective effects against oxidative stress-induced and glucocorticoid-induced muscle atrophies, which contribute significantly to muscle degeneration and dysfunction.

Dexa, an anti-inflammatory and immunosuppressive agent, is used clinically to manage various pathological conditions, including chronic obstructive pulmonary disease, asthma, rheumatoid arthritis, cancer, and allergic responses. However, prolonged high-dose Dexa administration may result in a range of adverse effects, particularly muscle weakness accompanied by reduced muscle mass [4,39]. Therefore, Dexa is frequently used to establish preclinical muscle atrophy models. Dexa can reliably induce muscle atrophy by suppressing the PI3K/Akt anabolic pathway and upregulating MuRF1- and Atrogin-1-mediated catabolic muscle degradation, which mimics clinical situations [40]. Therefore, we used experimental animals with Dexa-induced muscle atrophy to investigate the protective potential of GSP against glucocorticoid therapy-associated muscle loss.

Soybeans are an important ingredient in Korean cuisine and are considered a good source of vegetable proteins. They are rich in antioxidants, including anthocyanins, polyphenols, and flavonoids, which may reduce the risk of chronic diseases related to oxidative stress [41]. Bioconversion processes, such as soybean fermentation, can enhance the bioavailability of nutrients in soybeans and increase the content of isoflavone aglycones, including daidzein, glycitein, and genistein, compared to non-fermented soybeans. Isoflavone aglycones have higher antioxidant potential than isoflavone glucosides such as daidzin, glycitin, and genistin [42]. Other studies have shown that soybean fermentation increases anti-inflammatory properties by inhibiting the production of pro-inflammatory cytokines, nitric oxide, and prostaglandin E2 by lipopolysaccharide-activated immune cells [43], thereby relieving inflammatory pain in experimental mice. These findings suggest that bioconversion processes such as fermentation can boost the nutraceutical value of soybeans [43].

In this study, we evaluated the in vitro and in vivo protective effects of the ethanolic extract of GSP, which is prepared from fermented black soybeans, using Dexa-induced muscle atrophy models. The in vitro experiments demonstrated that GSP can protect undifferentiated C2C12 myoblasts (Figure 1) and differentiated C2C12 myotubes (Figure 2) from H_2_O_2_-mediated oxidative stress and cytotoxicity of Dexa at high concentrations, which induced muscle atrophy in C2C12 cells. The maintenance of myotube integrity was further supported by the preservation of MyHC expression, inhibition of muscle degradation-associated proteins such as Atrogin-1 and MuRF1, and upregulation of mitochondrial biogenesis. Such protective effects may be related to its antioxidant potential in terms of free radical-scavenging activity and decreased intracellular ROS production in C2C12 myoblasts and myotubes following GSP pretreatment and H_2_O_2_ treatment (Figure 3), which may be ascribed in part to the upregulation of antioxidant activity-related genes (Supplementary Figures S2 and S3). The muscle-protective potential of GSP was also supported by the downregulation of muscle degradation marker proteins MuRF1 and Atrogin-1 following H_2_O_2_-mediated oxidative stress challenge (Figure 2E). In addition, GSP itself enhanced the expression of genes related to myogenesis (*Myod1* and *Myog*) and mitochondrial energetics (*Pgc1α*) (Figure 1E and Supplementary Figure S1). MYOD1 and MYOG are transcription factors essential for the initiation and progression of myogenesis, while PGC1*α* is a crucial regulator of mitochondrial biogenesis and muscle fiber differentiation. The promotion of the expression of these genes by GSP indicates that it not only protects muscle cells from cytotoxicity but also enhances myoblast differentiation, a critical process in muscle regeneration. These findings suggest that GSP promotes muscle regeneration and repair under stressful conditions such as exposure to oxidative stress or glucocorticoids, contributing to muscle health maintenance and recovery.

Animal studies using mice with Dexa-induced muscle atrophy demonstrated that oral GSP administration significantly reduced both muscle wasting and dysfunction associated with the chronic Dexa toxicity (Figure 4). Although the effects of GSP co-administration were not statistically significant, GSP-treated mice showed improved motor coordination in the rotarod test, further supporting the hypothesis that GSP has a beneficial effect on muscle function under muscle atrophy. The muscle-protective effect of GSP was related to the restoration of muscle fiber integrity and downregulation of the muscle degradation marker proteins MuRF1 and Atrogin-1 (Figure 5), which are hallmarks of glucocorticoid-induced muscle atrophy. In addition, the proportion of fibers within the larger CSA range was significantly increased compared to that in the Dexa-only-treated group, whereas the proportion of smaller fibers decreased. This shift in muscle fiber distribution suggests that GSP helped to preserve larger muscle fibers, which are essential for maintaining overall muscle mass and strength. This protective effect of GSP is consistent with the results of previous studies reporting the beneficial effects of soybeans on muscle health. For example, soy protein supplementation has been shown to enhance the repair of damaged muscles, increase muscle growth, delay fatigue from high-intensity exercise, and enhance physical performance, which are all associated with improved lean body mass and reduced oxidative stress [44].

Oxidative stress, caused by an imbalance between ROS production and the antioxidant defense system of our body, can trigger NF-κB pro-inflammatory signaling pathways, which accelerate muscle protein degradation by activating ubiquitin–proteasome and autophagy–lysosomal pathways [45,46]. In experimental mice, chronic administration of Dexa resulted in the accumulation of oxidative stress markers and significantly induced the expression of the oxidative stress-sensitive ubiquitin ligase Cbl-b [47]. In contrast, the alleviation of oxidative stress successfully attenuates Dexa-induced muscle atrophy in experimental mice [48], suggesting the therapeutic potential of antioxidants in muscle atrophy induced by chronic glucocorticoid use. This is consistent with the results of the present study showing the muscle-protective effect of GSP via antioxidant activity against Dexa-induced muscle atrophy. Some isoflavones with antioxidant activity in soybeans prevent sarcopenic muscle loss by blocking the expression of MuRF1, a protein marker responsible for muscle degradation, and androgen receptors related to muscle mass growth [49]. Genistein and daidzein protect C2C12 myotubes from pro-inflammatory cytokine tumor necrosis factor-alpha-mediated myotube atrophy via upregulation of the AMP-activated protein kinase-Sirtuin 1 axis [50]. In our cell-free ABTS and DPPH free radical-scavenging assays, genistein (C9) showed higher scavenging activity against ABTS free radicals but similar activity against DPPH free radicals compared to its glycoside genistin. In contrast, daidzein (C7) showed lower scavenging activity against ABTS free radicals but similar activity against DPPH free radicals compared to its glycoside daidzin (C1) (Supplementary Figure S6), consistent with the results of a previous study [51]. However, daidzein showed comparable or higher antioxidant potential than daidzin in other antioxidant activity assays, such as the copper-chelating activity assay [52]. Other unidentified components in the GSP extract may also contribute to its antioxidant potential.

It is well known that isoflavones from soybeans, such as daidzein, genistein, and glycitein, may reduce muscle atrophy by blocking the expression of MuRF1 or by regulating androgen receptors [49,50]. To identify the components responsible for the promoter-enhancing activity of GSP related to myogenesis and mitochondrial bioenergetics (Figure 1E), nine individual GSP components were evaluated for their promoter-enhancing potential in C2C12 myoblasts. The results demonstrated that daidzin (C1), genistin (C3), and their aglycones daidzein (C7) and genistein (C9) exhibited greater potential in enhancing the activity of the *Myod1*, *Myog*, and *Pgc1α* promoters compared to other isoflavones (Figure 6D). The promoter-enhancing activity of these components was associated with their protective effects on C2C12 myotubes exposed to high doses of Dexa (Figure 6D). Myogenesis involves the activation and differentiation of quiescent satellite cells in response to muscle damage and therefore plays a key role in the repair process following muscle injury [53]. Additionally, mitochondrial dysfunction can accelerate muscle atrophy by limiting the energy supply and increasing ROS levels [54]. Therefore, enhancing the promoter activity of genes associated with myogenesis and mitochondrial energetics may help recover from or protect against muscle damage caused by chronic high-dose administration of glucocorticoids. Bioactive compounds from GSP, which significantly enhanced the promoter activity of *Myod1*, *Myog*, and *PGC1α*, reinforce the notion that flavonoids play a crucial role in mediating the beneficial effects of GSP on muscle health. Future studies should investigate the individual and synergistic bioavailabilities and pharmacokinetic contributions of these flavonoids to muscle preservation.

## 5. Conclusions

In summary, this study suggests that GSP effectively alleviates oxidative stress-induced and glucocorticoid-induced muscle atrophies. GSP exhibited muscle-protective effects in both C2C12 cells and animal models, indicating its potential as a supplementary agent for preventing and managing degenerative muscle conditions. The muscle-protective effects of GSP were attributed to its potential to relieve oxidative stress and enhance the expression of genes associated with myogenesis and mitochondrial bioenergetics. Consistent efforts in quality control, including standardization of harvesting and bioconversion of soybeans, are essential for maintaining the chemical composition of GSP raw materials. Further studies are required to elucidate the precise mechanism of GSP and evaluate its clinical applications.

## Figures and Tables

**Table 1:** Contents of the nine flavonoids in GSP according to HPLC–PDA assay (*n* = 3).

Flavonoid	RT (min)	Detection (nm)	Contents (mg/g)	RSD (%)
Daidzin	14.97	250	3.73	0.37
Glycitin	15.94	255	0.86	0.78
Genistin	20.03	260	8.07	0.36
Acetyldaidzin	24.39	250	0.26	1.26
Acetylglycitin	25.04	260	0.10	2.69
Malonylgenistin	25.47	260	0.15	2.36
Daidzein	28.12	250	1.57	0.39
Glycitein	28.97	255	0.21	1.42
Genistein	31.76	260	1.74	0.57

Abbreviations: RSD, relative standard deviation; RT, retention time.

## Data Availability

All data supporting the study findings are available within this paper.

## Supplementary Materials

The following supporting information can be downloaded at: https://www.mdpi.com/article/10.3390/antiox14070882/s1. Figure S1: Effects of GSP on gene expression related to myogenesis and mitochondrial biogenesis in C2C12 myoblasts. Figure S2: Effects of GSP on protein expression related to antioxidant activity in C2C12 myoblasts Figure S3: Effects of GSP on the expression of antioxidant-related genes in C2C12 myoblasts and myotube cells challenged with H2O2 treatment. Figure S4: Effects of GSP administration on the muscle weight in mice with Dexa-induced muscle atrophy. Figure S5: The effects of GSP on the myofibers and the expression of muscle degradation-related proteins in TA muscle in mice with Dexa-induced muscle atrophy. Figure S6: Analysis of free radical-scavenging activity.

## Abbreviations

The following abbreviations are used in this manuscript:

GSP	Glycine Semen Preparata
Dexa	dexamethasone
RsV	resveratrol
PDA	photodiode array detector
TA	tibialis anterior
GN	gastrocnemius
ROS	reactive oxygen species
CSA	cross-sectional area
HPLC	high-performance liquid chromatography

## References

[1] Yin L., Li N., Jia W., Wang N., Liang M., Yang X., Du G. (2021). Skeletal muscle atrophy: From mechanisms to treatments. Pharmacol. Res..

[2] Landi F., Camprubi-Robles M., Bear D.E., Cederholm T., Malafarina V., Welch A.A., Cruz-Jentoft A.J. (2019). Muscle loss: The new malnutrition challenge in clinical practice. Clin. Nutr..

[3] Evans W.J. (2010). Skeletal muscle loss: Cachexia, sarcopenia, and inactivity. Am. J. Clin. Nutr..

[4] Schakman O., Kalista S., Barbé C., Loumaye A., Thissen J.P. (2013). Glucocorticoid-induced skeletal muscle atrophy. Int. J. Biochem. Cell Biol..

[5] Anker S.D., Morley J.E., von Haehling S. (2016). Welcome to the ICD-10 code for sarcopenia. J. Cachexia Sarcopenia Muscle.

[6] Baek J.Y., Jung H.W., Kim K.M., Kim M., Park C.Y., Lee K.P., Lee S.Y., Jang I.Y., Jeon O.H., Lim J.Y. (2023). Korean Working Group on Sarcopenia Guideline: Expert Consensus on Sarcopenia Screening and Diagnosis by the Korean Society of Sarcopenia, the Korean Society for Bone and Mineral Research, and the Korean Geriatrics Society. Ann. Geriatr. Med. Res..

[7] Kim S., Ha Y.C., Kim D.Y., Yoo J.I. (2024). Recent Update on the Prevalence of Sarcopenia in Koreans: Findings from the Korea National Health and Nutrition Examination Survey. J. Bone Metab..

[8] Kwak J.Y., Kwon K.S. (2019). Pharmacological Interventions for Treatment of Sarcopenia: Current Status of Drug Development for Sarcopenia. Ann. Geriatr. Med. Res..

[9] Merz K.E., Thurmond D.C. (2020). Role of skeletal muscle in insulin resistance and glucose uptake. Compr. Physiol..

[10] Hargreaves M., Spriet L.L. (2020). Skeletal muscle energy metabolism during exercise. Nat. Metab..

[11] Gomarasca M., Banfi G., Lombardi G., Makowski G.S. (2020). Chapter Four—Myokines: The endocrine coupling of skeletal muscle and bone. Advances in Clinical Chemistry.

[12] Que Z., Jin Y., Huang J., Zhou R., Wu C. (2023). Flavor compounds of traditional fermented bean condiments: Classes, synthesis, and factors involved in flavor formation. Trends Food Sci. Technol..

[13] Qiao Y., Zhang K., Zhang Z., Zhang C., Sun Y., Feng Z. (2022). Fermented soybean foods: A review of their functional components, mechanism of action and factors influencing their health benefits. Food Res. Int..

[14] Chen J., Cheng Y.-Q., Yamaki K., Li L.-T. (2007). Anti-α-glucosidase activity of Chinese traditionally fermented soybean (douchi). Food Chem..

[15] Li T., Zhang X., Zeng Y., Ren Y., Sun J., Yao R., Wang Y., Wang J., Huang Q. (2023). Semen Sojae Preparatum as a traditional Chinese medicine: Manufacturing technology, bioactive compounds, microbiology and medicinal function. Food Rev. Int..

[16] Wang D., Wang L.J., Zhu F.X., Zhu J.Y., Chen X.D., Zou L., Saito M., Li L.T. (2008). *In vitro* and *in vivo* studies on the antioxidant activities of the aqueous extracts of Douchi (a traditional Chinese salt-fermented soybean food). Food Chem..

[17] Dwivedi S., Singh V., Sharma K., Sliti A., Baunthiyal M., Shin J.-H. (2024). Significance of soy-based fermented food and their bioactive compounds against obesity, diabetes, and cardiovascular diseases. Plant Foods Hum. Nutr..

[18] Fong F.L.Y., Lam K.Y., San Lau C., Ho K.H., Kan Y.H., Poon M.Y., El-Nezami H., Sze E.T.P. (2020). Reduction in biogenic amines in douchi fermented by probiotic bacteria. PLoS ONE.

[19] Shalal O.S. (2023). Anti-inflammatory activity of fermented soyabean (*Glycine max*) extract on macrophages by inhibiting cytokines expression. Gene Rep..

[20] Messina M., Bennink M. (1998). 10 Soyfoods, isoflavones and risk of colonic cancer: A review of the in vitro and in vivo data. Baillière’s Clin. Endocrinol. Metab..

[21] Liang C., Wang P., Li M., Li R., Lai K.P., Chen J. (2024). Anti-cancer mechanisms of natural isoflavones against melanoma. Heliyon.

[22] Ravindranath M.H., Muthugounder S., Presser N., Viswanathan S. (2004). Anticancer therapeutic potential of soy isoflavone, genistein. Adv. Exp. Med. Biol..

[23] Kim Y., Cho J.-Y., Kuk J.-H., Moon J.-H., Cho J.-I., Kim Y.-C., Park K.-H. (2004). Identification and antimicrobial activity of phenylacetic acid produced by *Bacillus licheniformis* Isolated from fermented soybean, Chungkook-Jang. Curr. Microbiol..

[24] Lee J.-S., Rho S.-J., Kim Y.-W., Lee K.W., Lee H.G. (2013). Evaluation of biological activities of the short-term fermented soybean extract. Food Sci. Biotechnol..

[25] Jayachandran M., Xu B. (2019). An insight into the health benefits of fermented soy products. Food Chem..

[26] Go J., Eun K.J., Hwa K.M., Kyoung K.E., Hwa S.S., Eun S.J., Sup K.D., Tae H.J., Hwang D.Y. (2016). Neuroprotective effects of fermented soybean products (Cheonggukjang) manufactured by mixed culture of *Bacillus subtilis* MC31 and *Lactobacillus sakei* 383 on trimethyltin-induced cognitive defects mice. Nutr. Neurosci..

[27] Hashimoto R., Sakai A., Murayama M., Ochi A., Abe T., Hirasaka K., Ohno A., Teshima-Kondo S., Yanagawa H., Yasui N. (2015). Effects of dietary soy protein on skeletal muscle volume and strength in humans with various physical activities. J. Med. Investig..

[28] Munekawa C., Okamura T., Majima S., River B., Kawai S., Kobayashi A., Nakajima H., Kitagawa N., Okada H., Senmaru T. (2024). Daidzein inhibits muscle atrophy by suppressing inflammatory cytokine- and muscle atrophy-related gene expression. Nutrients.

[29] Hashimoto Y., Okamura T., Bamba R., Yoshimura Y., Munekawa C., Kaji A., Miki A., Majima S., Senmaru T., Ushigome E. (2024). Miso, fermented soybean paste, suppresses high-fat/high-sucrose diet-induced muscle atrophy in mice. J. Clin. Biochem. Nutr..

[30] Kim A., Kim J., Kim B.-Y., Seo C.-S., Kim Y.R., Song K.H., Kim N.S. (2024). Aquo-ethanolic extract of Lilii Bulbus attenuates dexamethasone-induced muscle loss and enhances muscle strength in experimental mice. Biomed. Pharmacother..

[31] Ulla A., Uchida T., Miki Y., Sugiura K., Higashitani A., Kobayashi T., Ohno A., Nakao R., Hirasaka K., Sakakibara I. (2021). Morin attenuates dexamethasone-mediated oxidative stress and atrophy in mouse C2C12 skeletal myotubes. Arch. Biochem. Biophys..

[32] Kim A., Park S.-M., Kim N.S., Park M., Cha S. (2025). Ginsenoside Rc prevents dexamethasone-induced muscle atrophy and enhances muscle strength and motor function. J. Ginseng Res..

[33] Kim A., Kim Y.R., Park S.-M., Lee H., Park M., Yi J.-M., Cha S., Kim N.S. (2024). Jakyak-gamcho-tang, a decoction of Paeoniae Radix and Glycyrrhizae Radix et Rhizoma, ameliorates dexamethasone-induced muscle atrophy and muscle dysfunction. Phytomedicine.

[34] Rahmati M., Shariatzadeh Joneydi M., Koyanagi A., Yang G., Ji B., Won Lee S., Keon Yon D., Smith L., Il Shin J., Li Y. (2023). Resistance training restores skeletal muscle atrophy and satellite cell content in an animal model of Alzheimer’s disease. Sci. Rep..

[35] Lopez-Pedrosa J.M., Camprubi-Robles M., Guzman-Rolo G., Lopez-Gonzalez A., Garcia-Almeida J.M., Sanz-Paris A., Rueda R. (2024). The vicious cycle of type 2 diabetes mellitus and skeletal muscle atrophy: Clinical, biochemical, and nutritional bases. Nutrients.

[36] Murdock D.J., Wu N., Grimsby J.S., Calle R.A., Donahue S., Glass D.J., Sleeman M.W., Sanchez R.J. (2022). The prevalence of low muscle mass associated with obesity in the USA. Skelet. Muscle.

[37] Jun L., Robinson M., Geetha T., Broderick T.L., Babu J.R. (2023). Prevalence and mechanisms of skeletal muscle atrophy in metabolic conditions. Int. J. Mol. Sci..

[38] Bekircan-Kurt C.E., Subramanian S., Chagat S., Mackenzie S.J., Iammarino M., Reash N., Richardson C., Tsao C.Y., Noritz G., Gushue C. (2025). Transitioning from Nusinersen to Risdiplam for spinal muscular atrophy in clinical practice: A single-center experience. Muscle Nerve.

[39] Löfberg E., Gutierrez A., Wernerman J., Anderstam B., Mitch W.E., Price S.R., Bergström J., Alvestrand A. (2002). Effects of high doses of glucocorticoids on free amino acids, ribosomes and protein turnover in human muscle. Eur. J. Clin. Investig..

[40] Wang L., Jiao X.-F., Wu C., Li X.-Q., Sun H.-X., Shen X.-Y., Zhang K.-Z., Zhao C., Liu L., Wang M. (2021). Trimetazidine attenuates dexamethasone-induced muscle atrophy via inhibiting NLRP3/GSDMD pathway-mediated pyroptosis. Cell Death Discov..

[41] Hyun Young K., So-Yeun W., Ji Yeong Y., Seung-Yeob S., Woo Duck S., Mi Ja L., Man-Soo C. (2021). Antioxidant activities of various black soybean tissues (*Glycine max* L.) harvested from different cultivation regions. Korean J. Food Nutr..

[42] Jung T.-D., Shin G.-H., Kim J.-M., Oh J.-W., Choi S.-I., Lee J.-H., Lee S., Heo I., Park S., Kim H. (2016). Assessment of validation method for bioactive contents of fermented soybean extracts by bioconversion and their antioxidant activities. J. Korean Soc. Food Sci. Nutr..

[43] Yusof H.M., Ali N.M., Yeap S.K., Ho W.Y., Beh B.K., Koh S.P., Long K., Alitheen N.B. (2019). Anti-inflammatory, analgesic and acute toxicity effects of fermented soybean. BMC Complement. Altern. Med..

[44] Zare R., Devrim-Lanpir A., Guazzotti S., Ali Redha A., Prokopidis K., Spadaccini D., Cannataro R., Cione E., Henselmans M., Aragon A.A. (2023). Effect of soy protein supplementation on muscle adaptations, metabolic and antioxidant status, hormonal response, and exercise performance of active individuals and athletes: A systematic review of randomised controlled trials. Sports Med..

[45] Chen M., Wang Y., Deng S., Lian Z., Yu K. (2022). Skeletal muscle oxidative stress and inflammation in aging: Focus on antioxidant and anti-inflammatory therapy. Front. Cell Dev. Biol..

[46] Sandri M. (2013). Protein breakdown in muscle wasting: Role of autophagy-lysosome and ubiquitin-proteasome. Int. J. Biochem. Cell Biol..

[47] Ulla A., Osaki K., Rahman M.M., Nakao R., Uchida T., Maru I., Mawatari K., Fukawa T., Kanayama H.-O., Sakakibara I. (2022). Morin improves dexamethasone-induced muscle atrophy by modulating atrophy-related genes and oxidative stress in female mice. Biosci. Biotechnol. Biochem..

[48] Oh S., Choi C.H., Lee B.-J., Park J.-H., Son K.-H., Byun K. (2021). Fermented oyster extract attenuated dexamethasone-induced muscle atrophy by decreasing oxidative stress. Molecules.

[49] Lee S.-Y., Yoo J.-I. (2023). Soybean isoflavones potentially prevent sarcopenia: A systematic review. J. Ethn. Foods.

[50] Hirasaka K., Maeda T., Ikeda C., Haruna M., Kohno S., Abe T., Ochi A., Mukai R., Oarada M., Eshima-Kondo S. (2013). Isoflavones derived from soy beans prevent MuRF1-mediated muscle atrophy in C2C12 myotubes through SIRT1 activation. J. Nutr. Sci. Vitaminol..

[51] Lee C.H., Yang L., Xu J.Z., Yeung S.Y.V., Huang Y., Chen Z.-Y. (2005). Relative antioxidant activity of soybean isoflavones and their glycosides. Food Chem..

[52] Toda S., Shirataki Y. (2001). Comparison of antioxidative and chelating effects of daidzein and daidzin on protein oxidative modification by copper in vitro. Biol. Trace Elem. Res..

[53] Tsuchiya Y., Kitajima Y., Masumoto H., Ono Y. (2020). Damaged myofiber-derived metabolic enzymes act as activators of muscle satellite cells. Stem Cell Rep..

[54] Hyatt H.W., Powers S.K. (2021). Mitochondrial dysfunction Is a common denominator linking skeletal muscle wasting due to disease, aging, and prolonged inactivity. Antioxidants.

