# Supplementary material for: Ethanolic Extract of Glycine Semen Preparata Prevents Oxidative Stress-Induced Muscle Damage in C2C12 Cells and Alleviates Dexamethasone-Induced Muscle Atrophy and Weakness in Experimental Mice
Source: Antioxidants (Basel). 2025 Jul 18;14(7):882. doi: 10.3390/antiox14070882 (PMC12292089; doi:10.3390/antiox14070882)
Supplement: Supplementary file 1 [file antioxidants-14-00882-s001.zip › antioxidants-3659406-supplementary.pdf]

Supplementary Materials for

Ethanolic Extract of Glycine Semen Preparata Prevents Oxidative Stress Induced Muscle Damage in C2C12 Cells and Alleviates Dexamethasone Induced Muscle Atrophy and Weakness in Experimental Mice

Aeyung Kim[a,¶], Jinhee Kim[b,¶], Chang-Seob Seo[c], Yu Ri Kim[b],

Kwang Hoon Song[b,*], No Soo Kim[b,*]

* **Corresponding Author**: Kwang Hoon Song, Ph.D.; No Soo Kim, Ph.D.

This PDF file includes: Figure S1-S6

Quantitative real-time PCR

Total RNAs were isolated from C2C12 cells using an commercially available kit (#17221, iNtRON Biotechnology, Seongnam, Republic of Korea), and RNA concentration was measured using a NanoDrop 2000 spectrophotometer (Thermo Fisher Scientific). First-strand cDNA was synthesized from 1 μg total RNA and using the High-Capacity cDNA Reverse Transcription Kit (#4368814, Thermo Fisher Scientific). The qPCR was performed using a SYBR PCR master max (#4367659, Thermo Fisher Scientific), 25-fold diluted cDNA, and gene-specific primer pairs (final concentration: 250 μM, Genotech, Daejeon, Republic of Korea) on a CFX Real-Time PCR System (Bio-Rad). Relative gene expression was quantified using the $2^{-\Delta\Delta Ct}$ method, with normalization to the expression of *Gapdh* housekeeping gene. The sequences of gene-specific primer pairs and their corresponding reference sequences are as follows:

Gene-specific primer pairs used for qPCR.

**Targets**	**Forward (5′ → 3′)**	**Reverse (5′ → 3′)**	**Reference**
Cat	CGGCACATGAATGGCTATGGATC	AAGCCTTCCTGCCTCTCCAACA	NM_009804
Gapdh	AAGGTGGTGAAGCAGGCAT	GGTCCAGGGTTTCTTACTCCT	NM_008084
Gpx1	CGCTCTTTACCTTCCTGCGGAA	AGTTCCAGGCAATGTCGTTGCG	NM_008160
Hmox1	AGCCCCACCAAGTTCAAACA	CATCACCTGCAGCTCCTCAA	NM_010442
Nfe2l2	TCTTGGAGTAAGTCGAGAAGTGT	GTTGAAACTGAGCAAAAAAGGC	NM_010902
Nqo1	AGGATGGGAGGTACTCGAATC	AGGCGTCCTTCCTTATATGCTA	NM_008706
Ppargc1a	GAATCAAGCCACTACAGACACCG	CATCCCTCTTGAGCCTTTCGTG	NM_008904
Sod1	GGTGAACCAGTTGTGTTGTCAGG	ATGAGGTCCTGCACTGGTACAG	NM_011434

Figure S1

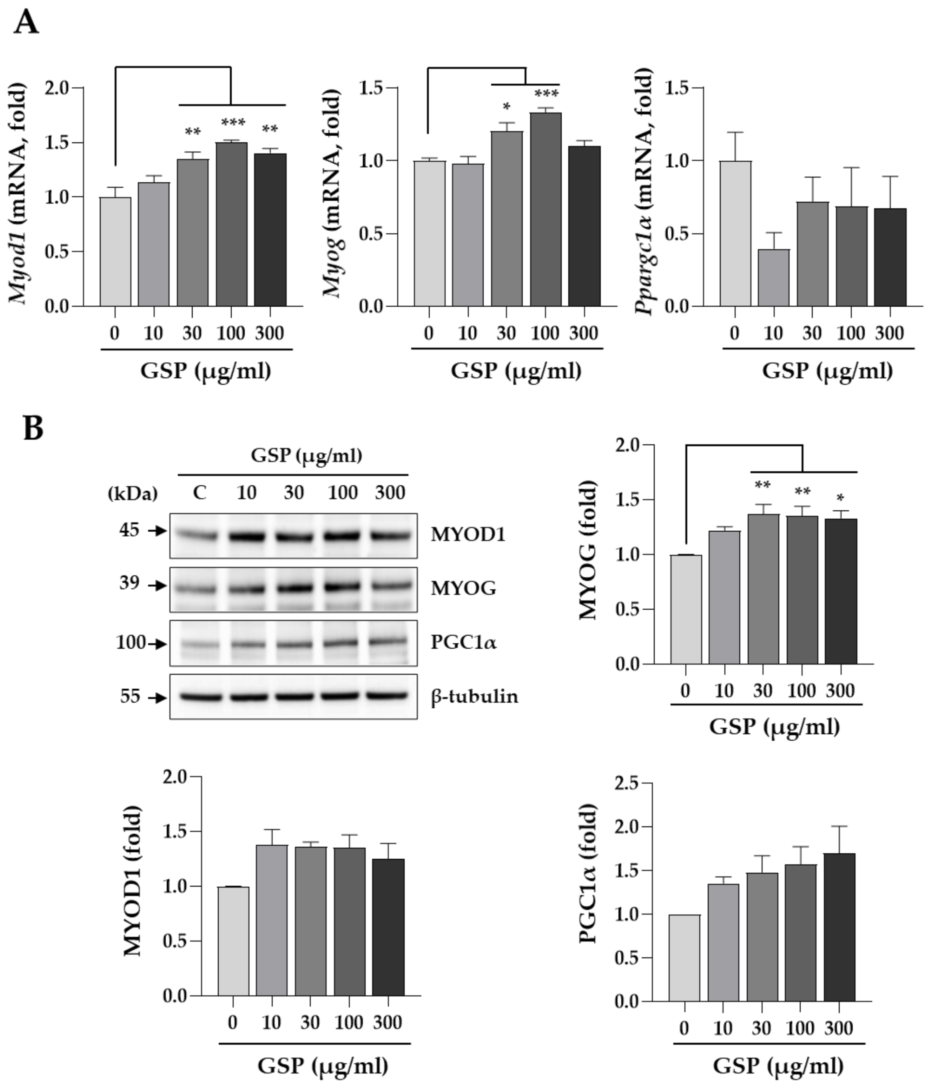

Figure S1. Effects of GSP on gene expression related with myogenesis and mitochondrial biogenesis in C2C12 myoblast. (A) Intracellular levels of *Myod1* and *Myog* mRNA were quantified in C2C12 myoblasts following 24 h-GSP treatment using qPCR. (B) Protein levels of MYOD1, MYOG, and PGC1α was assessed in C2C12 myoblast following 24 h-GST treatment by immunoblotting. The relative values are expressed as means ± SEM (n = 3). *p < 0.05, **p < 0.01, ***p < 0.001 vs. vehicle-treated controls.

Figure S2

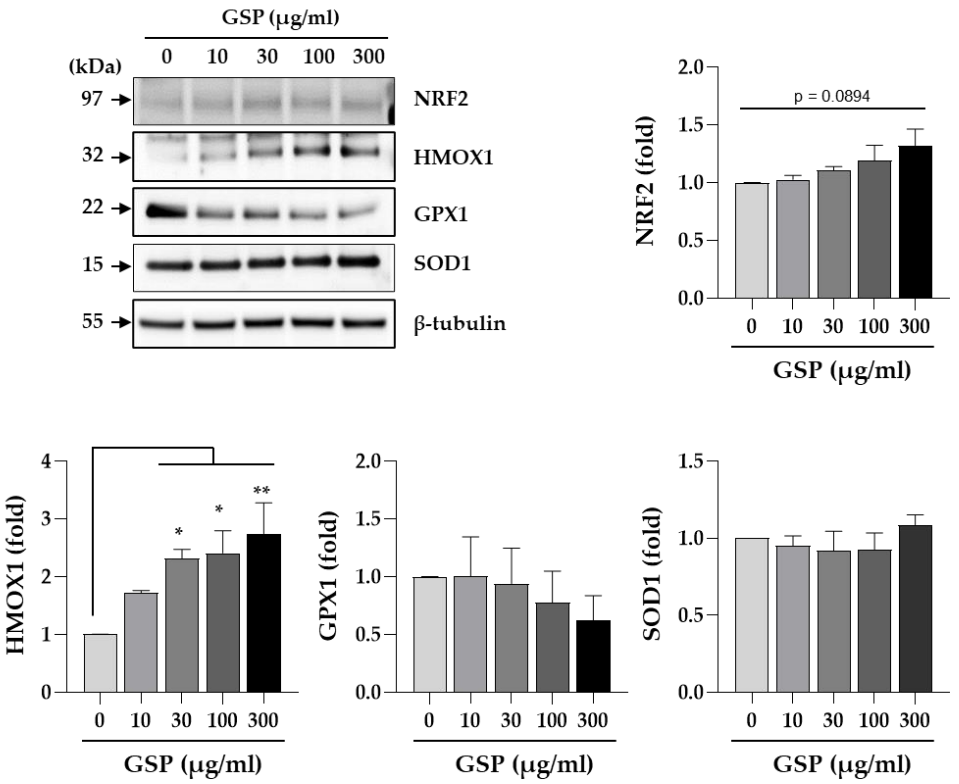

Figure S2. Effects of GSP on protein expression related with antioxidant activity in C2C12 myoblast. Protein levels related with antioxidant activity, such as NRF2, HMOX1, GPX1, and SOD1, was determined in C2C12 myoblast following 24 h-GSP treatment by immunoblotting. The relative values are expressed as means ± SEM ($n = 3$). *$p < 0.05$ and **$p < 0.01$ vs. vehicle-treated controls.

Figure S3

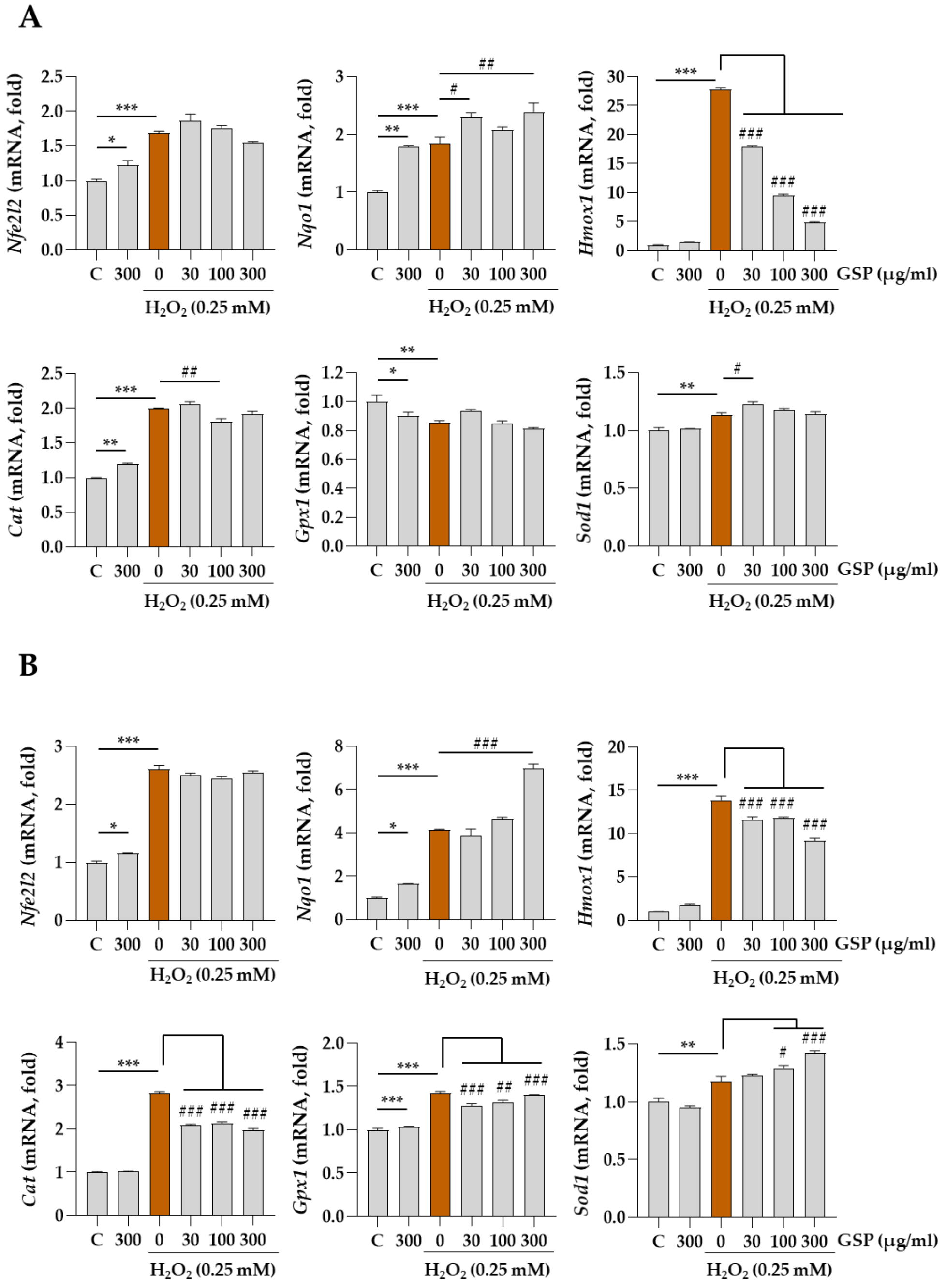

Figure S3. Effects of GSP on expression of antioxidant-related genes in C2C12 myoblast and myotube cells challenged with H_2O_2 treatment. Gene expression related with antioxidant activity were determined in C2C12 myoblast (A) or myotube (B). The cells were pretreated with GST for 12 h and then exposed to 0.25 mM H_2O_2 for 6 h (myoblast) or 24 h (myotube). Then, the intracellular mRNAs were quantified using qPCR. The relative values are expressed as means ± SEM (*n* = 3). **p* < 0.05, ***p* < 0.01, and ***p* < 0.01 vs. vehicle-treated controls. #*p* < 0.05, ##*p* < 0.01, ###*p* < 0.001 vs. H_2O_2 + vehicle-treated controls.

Figure S4

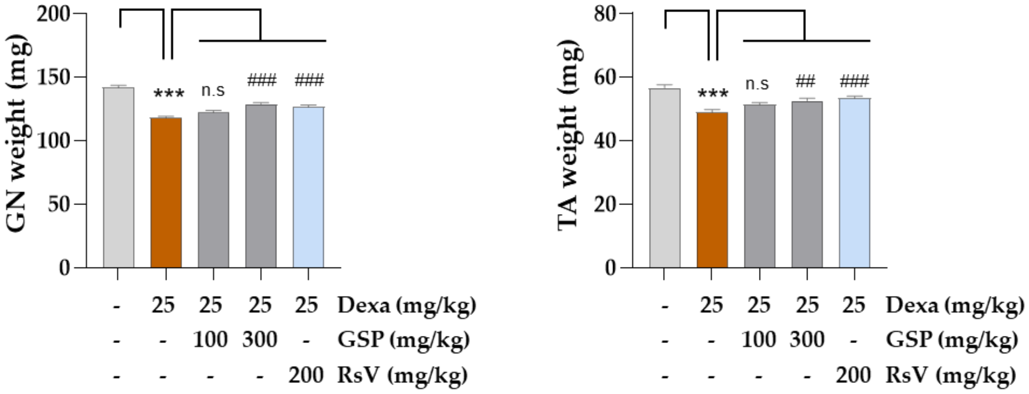

Figure S4. Effects of GSP administration on the muscle weight in mice with Dexa-induced muscle atrophy. On day 11, the gastrocnemius (GN) and tibialis anterior (TA) muscles were excised and weighed. Muscle weight was expressed as means ± SEM (n = 10). ***$p < 0.05$ vs. vehicle-treated group, ##$p < 0.01$, ###$p < 0.001$ vs. Dexa + vehicle-treated group. *n.s*, non-significant.

Figure S5

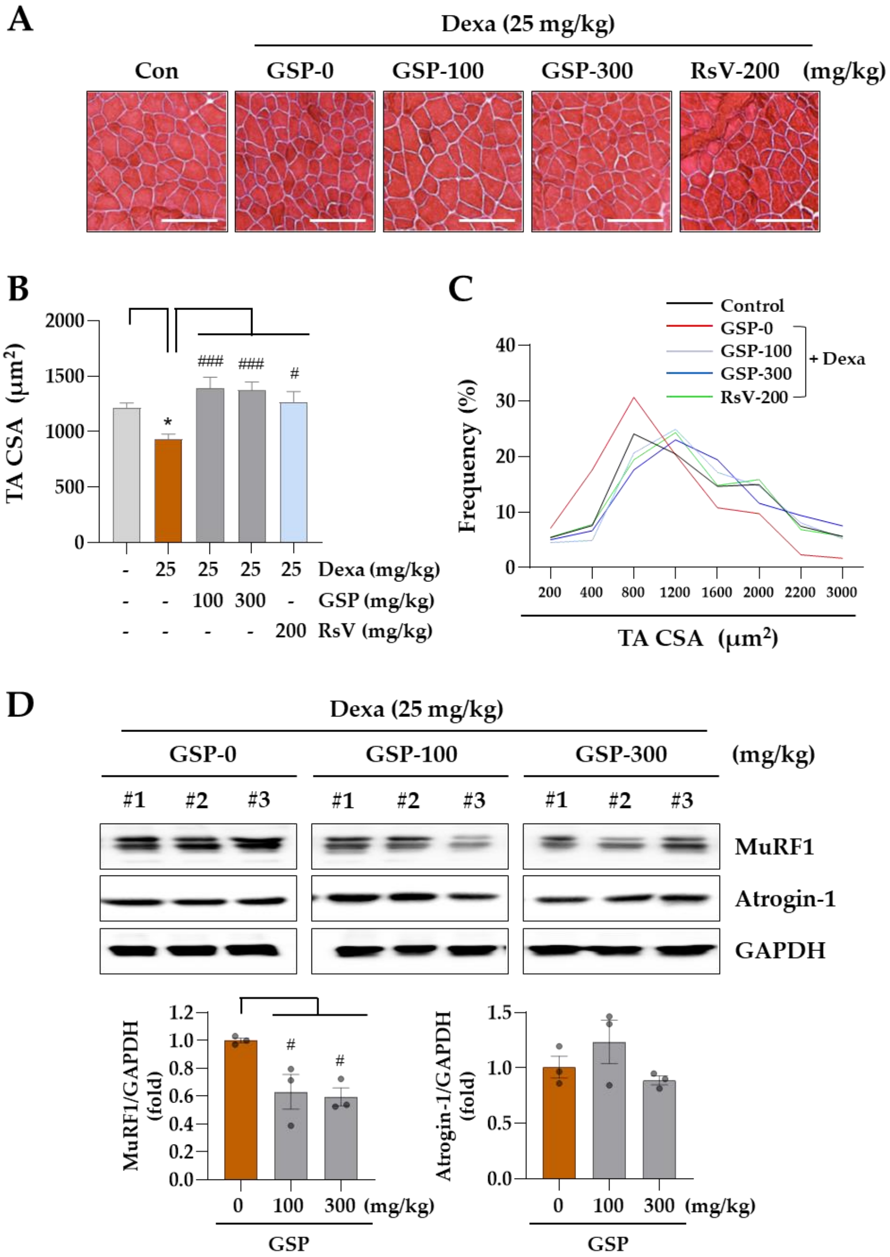

Figure S5. The effects of GSP on the myofibers and the expression of muscle degradation-related proteins in TA muscle in mice with Dexa-induced muscle atrophy. (A) After H&E staining, images for cross-sectional myofibers in TA muscle were presented. Scale bar = 100 μm. (B) The cross-sectional area (CSA) of the TA muscle fibers was quantified using ImageJ and expressed as means ± SEM ($n = 5$). (C) The frequency distribution of the CSA was analyzed. (D) The protein levels of MuRF1 and Atrogin-1 in TA muscles of mice were determined by immunoblotting ($n = 3$). GAPDH served as a loading control. *$p < 0.05$ vs. vehicle-treated group, #$p < 0.05$, ###$p < 0.001$ vs. Dexa + vehicle-treated group.

Figure S6

A

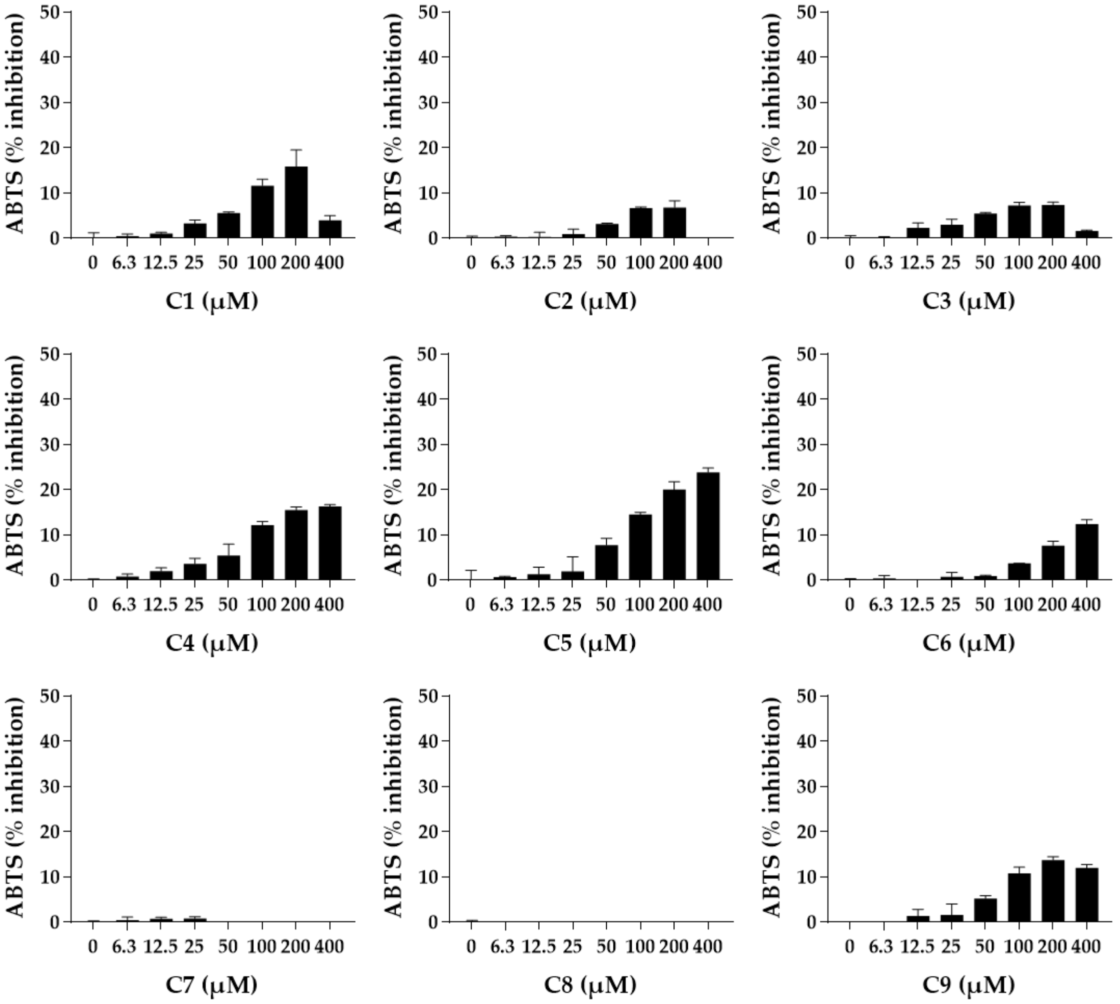
ABTS (% inhibition)
0
10
20
30
40
50
0
6.3
12.5
25
50
100
200
400
C1 (μM)
C2 (μM)
C3 (μM)
C4 (μM)
C5 (μM)
C6 (μM)
C7 (μM)
C8 (μM)
C9 (μM)

B

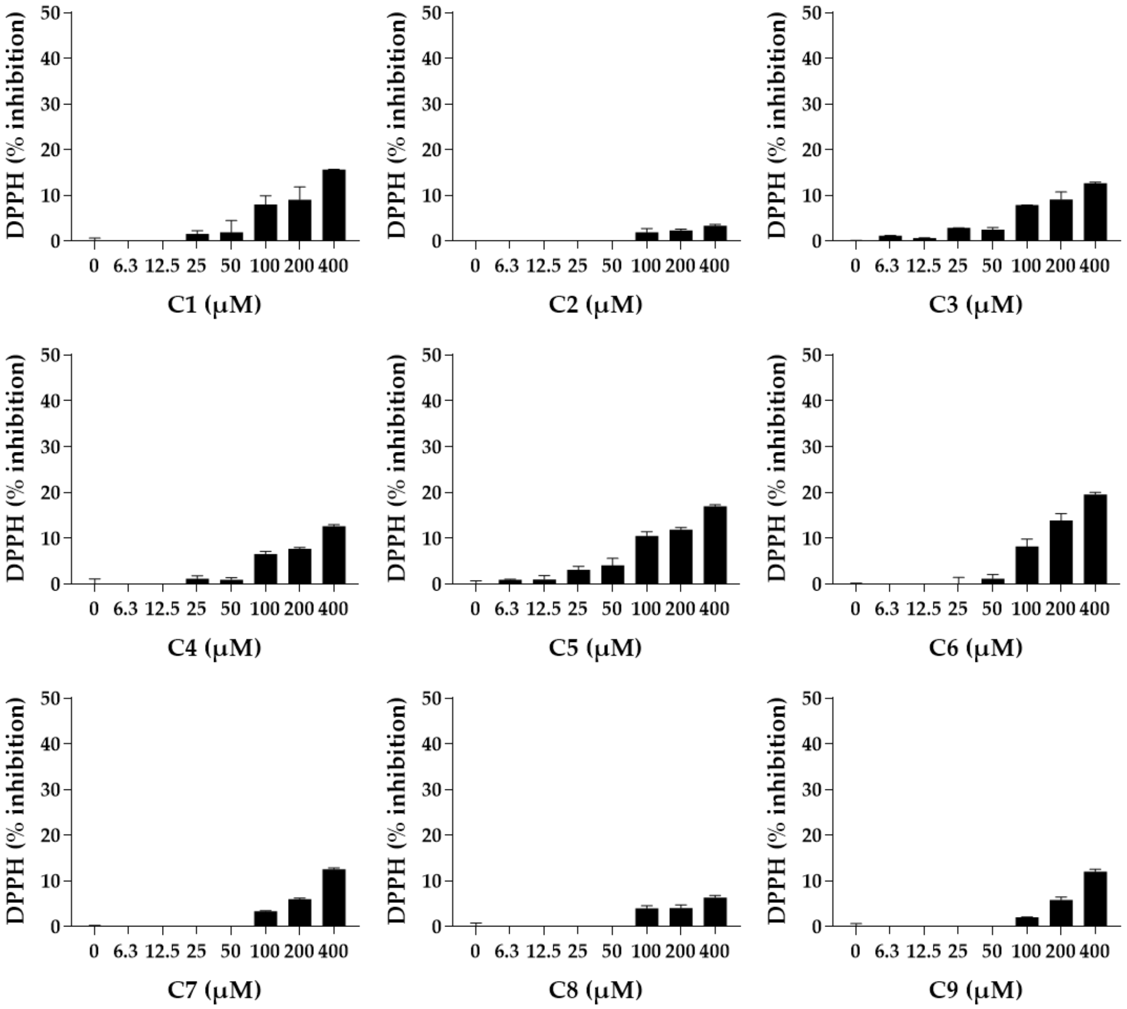

Figure S6. Analysis of free radial scavenging activity. Cationic and anionic free radical scavenging activities of flavonoids were determined using ABTS (A) and DPPH (B) assays, respectively. Data are expressed as means ± SD (n = 2).
